# Reciprocity, transitivity, and skew: Comparing local structure in 40 positive and negative social networks

**DOI:** 10.1371/journal.pone.0267886

**Published:** 2022-05-20

**Authors:** Cassie McMillan, Diane Felmlee, James R. Ashford

**Affiliations:** 1 Department of Sociology & Anthropology, School of Criminology & Criminal Justice, Northeastern University, Boston, MA, United States of America; 2 Department of Sociology & Criminology, Pennsylvania State University, University Park, PA, United States of America; 3 School of Computer Science and Informatics, Cardiff University, Cardiff, United Kingdom; Beihang University, CHINA

## Abstract

While most social network research focuses on positive relational ties, such as friendship and information exchange, scholars are beginning to examine the dark side of human interaction, where negative connections represent different forms of interpersonal conflict, intolerance, and abuse. Despite this recent work, the extent to which positive and negative social network structure differs remains unclear. The current project considers whether a network’s small-scale, structural patterns of reciprocity, transitivity, and skew, or its “structural signature,” can distinguish positive versus negative links. Using exponential random graph models (ERGMs), we examine these differences across a sample of twenty distinct, negative networks and generate comparisons with a related set of twenty positive graphs. Relational ties represent multiple types of interaction such as like versus dislike in groups of adults, friendship versus cyberaggression among adolescents, and agreements versus disputes in online interaction. We find that both positive and negative networks contain more reciprocated dyads than expected by random chance. At the same time, patterns of transitivity define positive but not negative graphs, and negative networks tend to exhibit heavily skewed degree distributions. Given the unique structural signatures of many negative graphs, our results highlight the need for further theoretical and empirical research on the patterns of harmful interaction.

## Introduction

Positive, cooperative ties among actors form the focus of numerous social network studies, with research on topics such as friendship, interlocking directorates, trade among nations, links within social media, kinship ties, and sexual relationships. Yet it remains without saying that negative, conflictual interconnections routinely characterize social interaction. A growing body of work brings attention to the importance of studying patterns of negative relationships (e.g., [1]). In negative social networks, connections are defined by harmful, anti-social sentiment, such as wagers of war between countries [2], physical violence among gangs [3], and instances of adolescent bullying and aggression [4]. Even though negative ties tend to be less common than positive links, the harm they inflict on actor-level outcomes and group-based processes is often disproportionate to the pro-social benefits of their positive counterparts (e.g., losing a friend versus gaining a friend) [5, 6]. People display a persistent “negativity bias,” in which they attend to negative stimuli and events more than the positive (e.g., [7, 8]).

While a great deal is known about the underlying, local structures that contribute to positive social graphs, research remains less informative regarding the basic features that compose less “sunny” networks. Pro-social ties tend to be reciprocated by actors who receive them [8, 9], and collegial relations are frequently defined by transitivity [10, 11], with actors connected to their connections’ connections. The practical implications of mutuality and transitivity, however, differ across graphs defined by positive versus negative valence [1] and thus, negative tie processes are unlikely to operate in identical ways to their positive counterparts [12]. Indeed, some previous work argues that the local structures known to dominate positive networks are less prevalent—or even non-existent—in negative graphs [13, 14], while others find that harmful networks exhibit these patterns with greater frequency [15, 16].

Given the serious problems associated with conflictual relationships in social life, it is important to gather systematic information regarding the composition of negative network ties. Here, we advance our understanding of the small-scale patterns that define negative social networks by comparing tendencies towards reciprocity, transitivity, and skewed degree distributions across a large, diverse sample of social graphs. Using exponential random graph models (ERGMs), we evaluate whether negative relations, such as dislike and bullying, exhibit similar local patterns as positive ties of friendship, amity, and cooperation. We investigate the structural components of twenty distinct, negative networks from a variety of on- and offline contexts and generate comparisons with a related set of twenty positive graphs. Examining multiple types of networks provides us with a relatively broad, cross-network view of local structure. As a result, we test whether negative networks develop “structural signatures” that distinguish them from their positive counterparts, while also considering whether patterns of negative ties differ across contexts.

## Background

As mentioned previously, there is an established body of work that considers the local patterns of networks defined by pro-social, collegial relationships (e.g., [10, 11]), while research on graphs defined by edges with negative sentiments is less common [1]. We next review previous empirical and theoretical literature on three structural components of interest: reciprocity, transitivity, and degree skew. In particular, we focus on how these structures may vary across positive and negative graphs. Then, we discuss how a comparative network approach is necessary when considering whether local patterns differ between these two types of graphs.

### Reciprocity

We begin by considering patterns of reciprocity, or mutual dyads where both actors recognize a social relationship (i.e., *a* → *b* and *b* → *a*). Many positive relationships are characterized by a “norm of reciprocity,” meaning that individuals feel obligated to repay previous favors and acts of kindness [17]. Social networks defined by amicable interactions are expected to include more reciprocated dyads than would be expected by random chance, even after accounting for various structural tendencies [8, 9]. Small-scale patterns of mutuality hold implications for higher order structures, such as triads [18] and tetrads [19], and reciprocity is a pervasive control in statistical models for positive graphs [20, 21]. In negative networks, patterns of reciprocity represent tendencies towards revenge, defensiveness, and retaliation [1]. Like their positive counterparts, there exists some empirical support that negative graphs are defined by more reciprocity than would otherwise be expected. For instance, gangs often seek revenge when targeted by rivals with gun violence [3] and adolescents frequently fight back when bullied by their peers [22, 23].

However, it remains unclear whether patterns of mutual dyads differ across positive versus negative social graphs. On the one hand, reciprocity could be more commonplace when connections are defined by negative, rather than positive, sentiment. The retaliation that defines patterns of reciprocity in negative graphs can result in self-perpetuating cycles of dislike that persist and intensify over extended periods of time. For example, Lerner and Lomi [16] find that when a Wikipedia user’s contribution is deleted by an adversarial user, the original user tends to react by deleting posts written by their rival. This results in an escalating cycle where negative reciprocal ties are highly stable, increasing the likelihood that mutuality will define a negative graph at any snapshot of time. Previous work on gossip networks also argues that patterns of revenge define the spread of damaging rumors. Ellwardt and colleagues [15] find higher levels of reciprocity in negative gossip networks—where ties indicate that the sender spread harmful gossip about the receiver—than in corresponding graphs of complementary gossip.

On the other hand, mutuality may be less ubiquitous in networks defined by negative interactions when compared to their positive counterparts [1]. Patterns of negative ties tend to reflect hierarchies of status. In some cases, this means that high-status individuals direct negative ties to lower-status peers who lack the necessary power to retaliate these attacks or may even return these harmful connections with positive links [13, 24]. More experienced players on an online gaming platform tend to execute attacks on newer, weaker players to ensure victory and avoid defensive assaults, for example [25]. In other situations, however, low-status actors extend negative ties to high-status individuals to reap the possible benefits associated with wounding a more socially advantaged peer [26, 27]. For instance, less acclaimed rap artists who release “diss songs” disparaging higher status rappers experience greater future sales than would be expected otherwise [28].

At the same time, negative relationships tend to be less visible than various types of positive connections. Social norms discourage openly admitting dislike or distrust of peers, and individuals are advised to avoid those with whom they hold negative sentiments [13, 29]. Graphs composed of negative ties also tend to be quite sparse, with relatively few edges connecting pairs of actors [12]. The low profile of these harmful connections should minimize opportunities for reciprocity to develop in negative relationships, particularly when compared to more visible and numerous positive connections. Some empirical work documents lower levels of mutuality in negative networks when compared to their positive counterparts. Among groups of children and adolescents, for example, bonds of friendship and esteem are more likely to be reciprocated than ties defined by dislike and animosity [13, 14, 30].

Given inconsistencies in the literature on the role of reciprocity in defining the patterns of negative relations, the current study considers whether tendencies towards mutuality vary in networks defined by positive versus negative connections (Research Question 1).

### Transitivity

In addition to considering the structural patterns of dyads, the social networks literature also has a long history of attention to triads, or groups of three actors (e.g., [10, 11, 31]). Informed by Heider’s [32] balance theory, research considers how local network structures are shaped by transitivity, or the tendency for two actors to be linked if they share a mutual connection (i.e., *a* → *b*, *b* → *c*, and *a* → *c*). Patterns of transitivity may help explain why networks are characterized by homophily [33, 34], and the prevalence of these configurations can inform macro-level structures of closure and hierarchy, simultaneously [11].

While previous work finds overwhelming evidence that transitivity defines the structures of positive relations, such as adolescent friendships, alliances between nation-states, and amicable interactions online [10, 35], it remains unclear how the phenomenon operates when negative ties are involved. In other words, is the enemy of one’s enemy also an enemy? This particular question remains controversial within the literature. Some studies find that transitivity need not define patterns of anti-social interaction [3, 23], while others argue that negative networks are characterized by more transitive triads than would be expected by random chance [2, 29].

There are reasons to expect that transitive triads will be less common in social networks defined by negative connections. Among positive interactions, triads that are not defined by transitive closure can result in higher levels of stress and cognitive dissonance for the individuals they connect [36]. Network members should have less awareness of these potentially stressful triads in negative interactions, however, because individuals tend to avoid those who they dislike [13]. Open, negative triads are unlikely to evoke dissonance and as a result, actors will have limited motivation to resolve these imbalances. For example, transitive triads are uncommon or even nonexistent in the aggression networks of young people [14], particularly when these harmful interactions occur in less visible mediums [22].

Another line of research argues that negative ties should exhibit the same tendencies towards transitive, triadic closure that are frequently observed in networks of amicable interaction. Given that local structures of transitivity suggest broader hierarchical patterns (e.g., [11]), there is also reason to believe that these triads will occur in negative graphs more frequently than expected. Negative ties often result from processes of status seeking and reinforcement [24, 37], which could produce patterns of transitivity. In such a case, actors on the receiving end of these triads (e.g., actor *c*, if *a* → *b*, *b* → *c*, and *a* → *c*) would be situated on the lowest rungs of the status hierarchy since they attract greater volumes of negative ties. Some empirical work finds support for these arguments. For instance, transitive triads appear more frequently than expected in networks of antagonistic ties among residents of a Honduran village [29], acts of dominance between summer campers [38], and harmful gossip links among coworkers [15].

Given these varying perspectives on triad patterns, the current project asks whether tendencies towards transitivity differ in networks defined by positive versus negative connections (Research Question 2). Specifically, we consider whether negative relations are more likely to connect a pair of actors if they also share a common, negative connection.

### Skewed degree distributions

The final local component we consider consists of individual actors’ patterns of sending and receiving ties, which culminate into distributions of outdegree (i.e., the number of sending ties) and indegree (i.e., the number of receiving ties). When connections are defined by positive, pro-social interaction, their distribution among actors tends to be skewed such that most individuals send and receive relatively modest numbers of ties, while a small handful report numerous connections. These disparities are often attributed to the social phenomenon of preferential attachment, which is also known as the “rich getting richer” or the Matthew effect, and can explain why well-embedded people become even more connected over time [39]. Previous work on pro-social networks finds evidence for skewed degree distributions within networks of adolescent friendship [13], sharing links on social networking websites [40], and transactional networks of Bitcoin addresses [41]. While actors who report many positive ties are typically considered to be well-liked, popular, or social, being highly embedded in a network of negative links carries different connotations. Receiving many negative ties suggests that an actor is disliked or low status, while sending large numbers of harmful ties indicates characteristics such as disagreeability, aggression, or antagonism.

Given that connectivity holds different implications in positive and negative relationships, there is reason to believe that negative graphs will exhibit degree distributions that differ from their positive counterparts in one of two ways. On the one hand, negative networks may be characterized by more uniform degree distributions than their positive counterparts. Actors tend to have a limited understanding of the hierarchical structures that define negative interactions since many of these connections are unobservable and the underlying network is often ambiguous [6, 13]. Without this knowledge, preferential attachment will be impeded because group members have less awareness of who is already well-connected.

On the other hand, previous empirical work finds significantly greater skew in the degree distributions of negative networks when compared to corresponding graphs of positive connections (e.g., [25, 30]). These findings reflect the fact that social norms and the desire for group cohesiveness encourage most actors to avoid negative interaction. Only a small minority of group members are actively involved in negative relations, resulting in a handful of “scapegoats” and “bad apples.” Scapegoats are typically low-status actors who receive disproportionately high numbers of negative ties [15], while bad apples send many of these links, perhaps because of traits that encourage their participation in aggressive, anti-social behaviors [6].

To better understand how processes like preferential attachment shape the formation of negative networks, we test whether degree distributions vary in networks defined by positive versus negative connections (Research Question 3). Specifically, we examine differences in the distributions of actors’ indegree and outdegree. Next, we discuss how we can address our three research questions regarding the local structures of positive and negative graphs by adopting a comparative approach that considers numerous networks from distinct genres of social interaction.

### A comparative approach

Although there has been a notable increase in research on negative ties in the past decade, much of this work analyzes single, empirical networks (e.g., [13, 15]). When multiple networks are considered, scholars tend to focus on one genre of negative relations, such as bullying among adolescents [4] or antagonistic ties among adult villagers [29]. Previous comparative network research demonstrates that there is value in studying patterns of interaction across diverse types of collectives [18, 19, 42, 43]. For instance, many of the same local structures characterize positive networks of email sending, patterns of U.S. senate co-sponsorship, and advice giving among coworkers [19].

It is also important to study negative networks in their own right, as opposed to focusing only on the ways they shape and are shaped by positive relations [13, 23]. Here, we conceptualize patterns of positive and negative ties as distinct networks to uncover new insight about the local properties that structure networks defined by disagreement and hostility. Direct comparisons between these two types of graphs can inform the development of theory on harmful, anti-social relationships, particularly when structural patterns differ according to a network’s valence. For example, there is debate over what comprises a negative tie in social networks because many relationships carry simultaneous benefits and costs (e.g., “the dark side of social capital,” [44]). If certain local structures are more (or less) likely to define negative networks than their positive counterparts, these patterns could encourage theoretical development on what should and should not constitute a negative tie. Additionally, researchers could use these findings to establish the sentiment of graphs where ties are characterized by ambiguous affect. One could determine whether a network of teasing among adolescents is defined by friendly horseplay or harmful aggression by evaluating only the graph’s structural characteristics, for example.

In the current project, we take a comparative approach by considering a sample of distinct positive and negative networks from five genres of social interaction. We compare patterns of reciprocity, transitivity, and skewed in- and outdegree distributions to enhance our ability to identify important commonalities and differences across social graphs. Every negative network in our sample is accompanied by a corresponding positive graph that contains identical set of actors at the same point in time. This enables us to test whether we can distinguish between networks defined by positive versus negative sentiment by solely considering patterns of local structures (Research Question 4).

Similar to how reciprocated dyads and transitive triads make up the building blocks of positive networks [9], there may be fundamental local structures that define patterns of negative interaction. At the same time, comparative network research is useful for highlighting the ways that structural patterns differ across diverse types of negative interaction [1, 23]. For instance, harmful interactions that occur in highly visible, online settings are more tractable than sentiments of dislike or distrust that exist offline. The costs and rewards of forming a negative link may carry heightened salience in these more visible contexts, and therefore influence patterns of local structure. Although we search for common structural patterns across our sample of negative networks, we also expect that some notable variations will surface between genres. We adopt a comparative network approach to identify when these discrepancies occur and to develop more insight into why these structural variations exist.

## Methods

### Data

**Fig 1:**
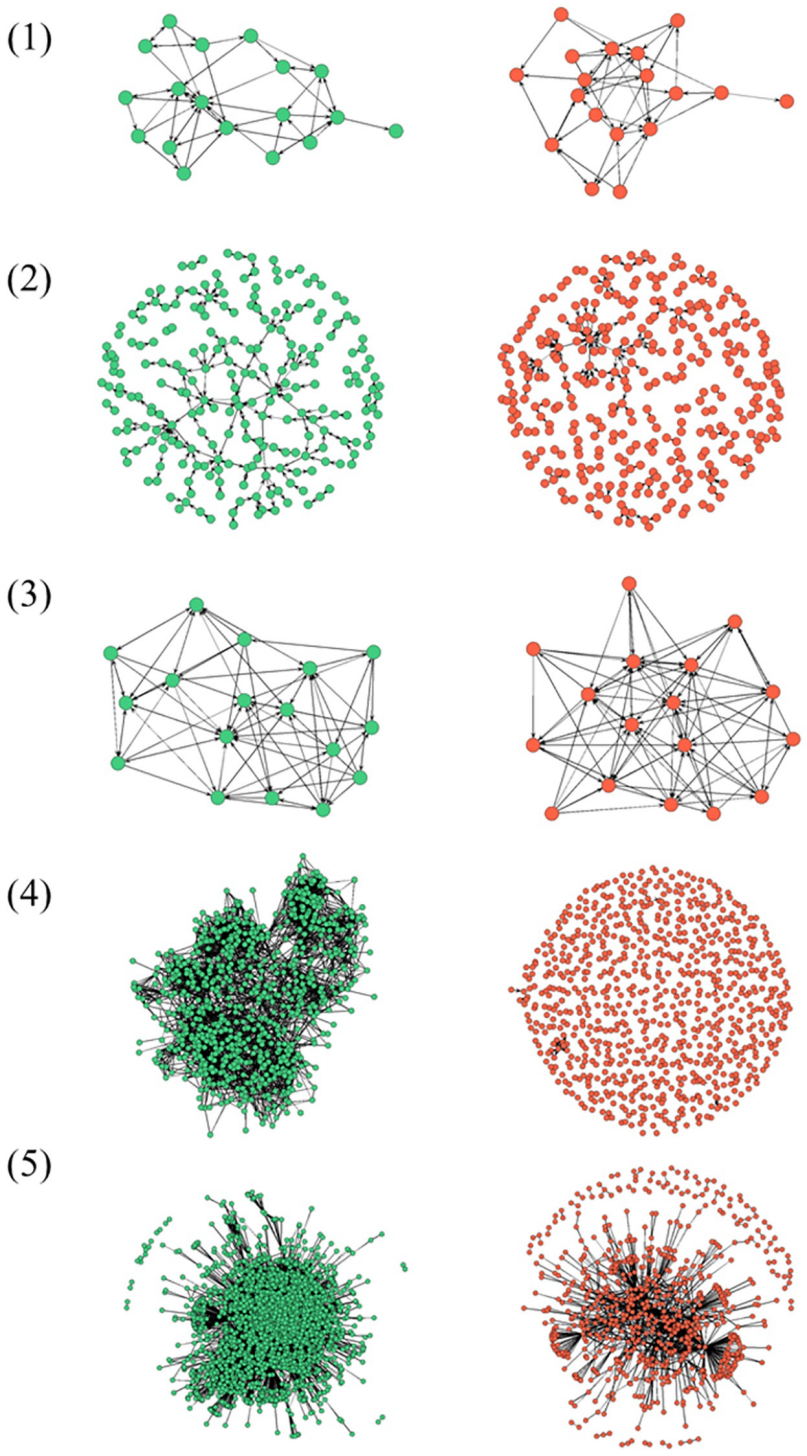
Examples of corresponding positive and negative graphs. Networks defined by positive sentiment are defined by green nodes. Red nodes indicate negative social networks. Network examples are from (1) Sampson’s Monastery, (2) Wikipedia editing, (3) Newcomb’s fraternity, (4) Friendship and bullying in a US high school, and (5) Ratings of trust on a Bitcoin trading platform. Isolated nodes are removed in several graphs for the purpose of clearer visualizations.

We consider local structures across forty networks that represent various types of interpersonal interaction. Twenty of these networks include connections defined by negative, harmful, or antagonistic sentiment, while the remaining twenty graphs contain positive relationships that occur among the exact same sets of actors. Our sample of networks can be organized into five genres: (1) amicable and antagonistic relationships among a sample of monks residing in a monastery, (2) complementary and disagreeable editing of Wikipedia articles, (3) most and least liked housemates in a college fraternity, (4) friendship and cyberbullying in a US high school, and (5) trust and distrust on an online currency trading platform. For a sample of several of our positive and negative graphs, see Fig 1. We focus on these five network types because they vary on key dimensions that are apt to shape local network patterns, such as whether interactions occurred on- or offline and the degree to which ties are publicly visible. Acts of Wikipedia editing and ratings of trust on an online currency trading platform, for example, are more transparent and distinct than the sentiments of like and dislike in fraternities, schools, and monasteries.

Our networks of positive and negative interactions between monks living in a monastery are constructed using Sampson’s classic monastery data [45]. Surveys were administered to an incoming class of eighteen monk novitiates that asked about their relationships to cohort-mates. Each monk nominated those peers with whom they maintained four types of positive connections (esteem, like, praise, and see as a positive influence) and four corresponding negative relationships (disesteem, dislike, blame, and see as a negative influence). Respondents were able to nominate up to three peers for each relationship type. Using this nomination data, we constructed eight directed networks where nodes are individual monks and a tie from node *a* to node *b* indicates that monk *a* nominated monk *b* for a survey item.

We constructed positive and negative networks from data on the editing patterns of four randomly selected “controversial” articles on Wikipedia, an open-source, online encyclopedia that allows users to make edits to each other’s posts on various informational topics [46]. Wikipedia users designate certain articles as controversial because they are the focus of frequent editing. We consider random controversial articles here because the high levels of editing activity ensured that our networks had sufficient densities to be analyzed with statistical network models. In the resulting eight networks, nodes represent the set of all users who edited the page of interest. A directed edge connects editor *a* to editor *b* if editor *a*’s edits follow those of editor *b* in the revision log. We infer that these directed edges are negative if the change in file size from one revision to another is less than zero, suggesting that editor *a* deleted editor *b*’s previous work. Alternatively, when the change in file size is greater than zero, we label these connections as positive ties since this activity implies that user *a* made a collaborative addition to user *b*’s post.

The networks of most and least liked fraternity brothers are constructed from Newcomb’s classic fraternity dataset [47]. Seventeen men living in a fraternity took weekly surveys where they were asked to rank all their fellow fraternity brothers starting from their first sociometric preference and ending with their last. Following Skvoretz and Faust [43], we conceptualize each respondent’s top five preferences as positive ties and their bottom five as negative connections. We consider eight waves of the data, resulting in a total of sixteen networks where nodes are fraternity brothers and directed edges indicate that brother *a* rated brother *b* as one of their top or bottom preferences.

Using data from the Long Island Study, we constructed networks of friendship and cyberaggression among nearly 800 students enrolled in grades eight through twelve at a public high school in New York [22]. Students were administered questionnaires during the school year that asked them to report up to ten of their closest friends, up to eight students “who picked on you or were mean to you,” and up to eight peers who “you picked on or were mean to” over the previous week. For both survey items about aggression, respondents were asked whether the behavior occurred online or over text message, allowing us to distinguish specific instances of cyberaggression. Using students’ friendship nominations, we constructed positive networks where a directed edge from student *a* to student *b* indicates that student *a* nominated student *b* as a friend. For the negative networks, we collapsed respondents’ nominations of cyberaggression victims and perpetrators, such that a directed edge from student *a* to student *b* suggests that student *a* cyberbullied student *b*. We considered patterns of positive and negative interaction at two periods of time, resulting in a total of four networks.

Finally, we constructed networks of trust and distrust from user ratings on Bitcoin OTC, an online trading platform for the cryptocurrency Bitcoin [48, 49]. The trading platform maintains a record of each user’s reputation by asking members to rate their transactions with one another on a scale from -10 to 10. A score of negative ten represents the greatest level of distrust, while a score of positive ten indicates complete trust. By considering all user ratings during two calendar years (2012 and 2013), we construct a total of four networks where nodes represent individual users on the trading platform. In the two positive trust networks, a directed tie indicates that user *a* gave user *b* a positively valued trust score, while a tie from user *a* to user *b* represents a negatively valued trust rating in the distrust graphs.

Our analyses of these secondary data sources were approved by the Pennsylvania State University IRB (STUDY00008769) and the University of California, Davis IRB (IRBNet #252367). We also complied with the terms of service for all websites and databases from which we collected data.

### Plan of analysis

To compare local patterns between our samples of positive and negative networks, we followed a plan of analysis that consisted of three steps. First, we estimated exponential random graph models (ERGMs) on each of our forty networks that included parameters to measure tendencies towards reciprocity, transitivity, and skew. Next, we used the standardized coefficients from each ERGM to estimate a set of predicted probabilities that a tie will connect each dyad. Finally, we used these predicted probabilities to calculate correlation coefficients between every pair of networks in our sample to compare the graphs systematically.

#### Step 1. Estimate ERGMs

To quantify the extent to which our networks are characterized by local patterns of interest, we first estimated ERGMs on each of the forty graphs in our sample. ERGMs represent a statistical network method that can determine whether an observed network’s structural patterns significantly differ from what would be expected to occur randomly [21, 50]. If we define an observed network as an *n* × *n* matrix (where *n* equals the number of actors), or **Y**, then each (*i*, *j*) entry will equal 1 if actors *i* and *j* are connected by a relational tie and 0 otherwise. The ERGM estimates the probability that **Y** will occur, given a set of network actors:

$$\begin{array}{c} P\left(Y=y|X\right)=\frac{\text{exp}[\theta^{T}g\left(y\right)]}{k(\theta )} \end{array}$$ (1)

All included covariates are presented in matrix **X** and *θ* is a vector of coefficients that are hypothesized to shape tie patterns in the observed network. Using the observed adjacency matrix, we can calculate a vector of network statistics, *g*(*y*), while *k*(*θ*) serves as a normalizing factor to ensure that we are predicting a legitimate probability distribution.

We included a total of six parameters in our ERGMs to compare various local patterns across our samples of positive and negative networks. First, all ERGMS included an *edges* term to account for the likelihood that an edge will exist between any pair of actors in a network. The edges term plays a similar role as the intercept in standard regression models and is expected to produce large, negative coefficients in all but the densest networks. To account for tendencies towards mutuality, we included a *reciprocity* term that counts the number of dyads where actor *i* sends a tie to actor *j* and actor *j* sends a tie to actor *i*.

Next, we included a set of two, commonly used terms to account for local triadic structures and measure transitivity across our networks. The first is known as the *geometrically weighted dyad-wise shared partner (GWDSP)* term and the second is the *geometrically weighted edge-wise shared partner (GWESP)* term. The GWDSP parameter accounts for the tendency for actor pairs to be linked through at least one mutual connection, regardless of whether the dyad is connected directly. The GWESP term considers only the degree to which connected actors share common partners in the network. Each variable is assigned a decay parameters that determines how much every additional shared connection contributes to the measure. For the current project, we use the GWESP coefficient to measure tendencies towards transitivity, while the GWDSP parameter is included to reduce the bias of these estimates [51, 52].

We also incorporate the *geometrically weighted outdegree distribution (GWO)* and *geometrically weighted indegree distribution (GWI)* terms to account for skew in actors’ patterns of sending and receiving ties. The GWO term adds the observed network’s outdegree distribution as a network statistic after weighting it according to a user-specified decay term. The GWI term is constructed in a similar manner, except it considers the distribution of actors’ indegree [53]. Negative values of the GWO and GWI coefficients are understood to suggest heavily skewed distributions in which only small numbers of actors either receive or send large numbers of ties. Alternatively, positive values of either coefficient suggest that patterns of receiving or sending ties are more uniform among a network’s actors.

To achieve satisfactory convergence across all the ERGMs we estimated, we needed to exclude some of the parameters discussed above for models estimated on certain networks (following [43]). Many of these exclusions result from varying data collection strategies. For instance, the Newcomb fraternity networks are constructed such that all actors have an outdegree of five, and this lack of variation makes it impossible to estimate models that include GWO terms on these graphs. For other networks, it was not possible to estimate ERGMs that included all six parameters because of problems with degeneracy. In these cases, we estimated reduced ERGMs that included various combinations of the four or five parameters that best fit the observed data. Even with these omissions, all ERGMs presented here include at least four parameters that account for micro-level, structural properties, with 78% of models including five or more parameters. Details on the included terms, associated decay parameters, goodness of fit, and other diagnostic tests are available in S1 File. (Parts B and C).

#### Step 2. Calculate predicted probabilities

Next, we used a set of coefficients and change scores from each estimated ERGM to calculate a series of predicted probabilities for every observed network (following [42, 43]). In the findings presented here, the set of coefficients does not include the value for the edges term (following [42]), but results are substantively similar when these coefficients are included. Each individual predicted probability refers to the odds that a tie will connect a specific actor dyad in an observed network. To make cross-network comparisons, we calculated these predicted probabilities for all actor pairs in each observed network using the ERGM coefficients for the focal graph, as well as those estimated on each of the other 39 networks. For example, we calculated 40 sets of predicted probabilities for the dislike network from Sampson’s monastery study. One set used coefficients from the ERGM estimated on this dislike network itself, while the other 39 sets considered the estimated ERGM coefficients for each of the other networks in our sample. This resulted in 1600 sets of predicted probabilities, with 40 sets for each of the 40 networks in our sample.

#### Step 3. Correlation coefficients

By comparing different networks’ sets of predicted probabilities, we can determine whether two networks share similar local structures. For instance, if a cyberbullying network is defined by similar patterns as the dislike network from Sampson’s monastery study, then using the cyberbullying network’s ERGM coefficients should result in a similar set of predicted probabilities as those calculated with the dislike network’s ERGM coefficients. We make these comparisons by calculating Pearson’s correlation coefficient for each set of predicted probabilities:

$$\begin{array}{c} r\left(f,y\right)=\frac{\sum \left(x_{f}\left(i,j\right)-m_{f}\right)\left(x_{y}\left(i,j\right)-m_{y}\right)}{\sqrt{\sum {\left(x_{f}\left(i,j\right)-m_{f}\right)}^{2}\sum {\left(x_{y}\left(i,j\right)-m_{y}\right)}^{2}}} \end{array}$$ (2)

Here, *x*_*f*_(*i*, *j*) is the predicted probability that node *i* will send a directed tie to node *j* in the focal network *f*, as predicted by the ERGM estimated on observed network *f*. Similarly, *x*_*y*_(*i*, *j*), represents the predicted probability that a tie will link node *i* to *j* in focal network *f* but as predicted by the ERGM estimated on observed network *y*. Both *m*_*f*_ and *m*_*y*_ represent the mean value of all (*i*, *j*) pairs’ predicted probabilities, which are calculated according to the ERGM coefficients for observed networks *f* and *y*, respectively. Higher values of *r*(*f*, *y*) indicate that a pair of networks are defined by similar tendencies toward reciprocity, transitivity, and skewed in- and outdegree distributions. We also calculated dissimilarity scores between our networks, using Euclidean distance (following [42]), and results are substantively similar to those presented here.

## Results

### ERGM findings

We uncovered notable similarities and differences across the local patterns of positive versus negative networks in our sample. To summarize our findings, we present two meta-analyses in Table 1 where the ERGM coefficients for the positive and negative networks are averaged separately, and then weighted according to the precision of each coefficient’s standard error (see S1 File, Part A for individual ERGM results). First, we found that patterns of reciprocity define the structures of positive and negative interaction at similar magnitudes after accounting for all other parameters included in our models *(b* = 2.70, *p* < 0.001 and *b* = 2.65, *p* < 0.001, respectively) (Research Question 1). These findings suggest that actors reciprocate social connections far more frequently than would be expected by random chance, regardless of whether ties are defined by friendly or harmful interaction.

**Table 1:** Meta-analyses of ERGM coefficients by network sentiment.

	Positive Networks			Negative Networks		
	*b*	SE		*b*	SE	
Reciprocity	2.696	(0.486)	***	2.647	(0.453)	***
GWESP (Transitivity)	1.053	(0.179)	***	0.127	(0.246)	
GWDSP	-0.240	(0.026)	***	-0.037	(0.065)	
GW Indegree	-0.394	(0.247)		-3.152	(0.622)	***
GW Outdegree	-0.504	(0.647)		-1.531	(0.466)	**
Edges	-3.379	(0.612)	***	-2.751	(0.661)	***
*N*	20			20		

** *p* < 0.01,

*** *p* < 0.001.

Robust standard errors are reported.

At the same time, there are also notable differences between the local structures of the positive and negative networks in our sample. Among the positive networks, there is a statistically significant tendency towards transitivity (*b* = 1.05, *p* < 0.001), while the negative networks tend to be defined by similar levels of transitive triads as would be expected to occur by random chance (*b* = 0.13, *p* = 0.61) (Research Question 2). If actor *i* sends a bond of affinity to actor *j* and actor *j* sends such a tie to *k*, then actor *i* is roughly 2.9 times more likely to send a positive tie to actor *k* than would otherwise be expected. However, when connections are defined by negative sentiment, we found that there is no statistically significant tendency towards transitive, triad closure, on average. Actors do not necessarily display the same sentiments of dislike towards third parties as those expressed by peers they dislike, for example.

Additionally, we found that our sample of negative networks tends to be characterized by in- and outdegree distributions that are more skewed than those of our positive networks, even after accounting for the other local patterns included in our models (Research Question 3). On average, the coefficients for both degree distribution parameters are negative and significant when networks are defined by antagonistic connections (GWO: *b* = -1.53, *p* < 0.01; GWI: *b* = -3.15, *p* < 0.001). These findings suggest that most actors send and receive few, if any, negative ties. Only a small minority are responsible for sending large volumes of negative ties, and an even smaller minority are the targets of these attacks. Among our positive networks, the average coefficients for the two skew parameters are negative, but they do not achieve statistical significance (GWO: *b* = -0.50, *p* = 0.45; GWI: *b* = -0.39, *p* = 0.13).

**Fig 2:**
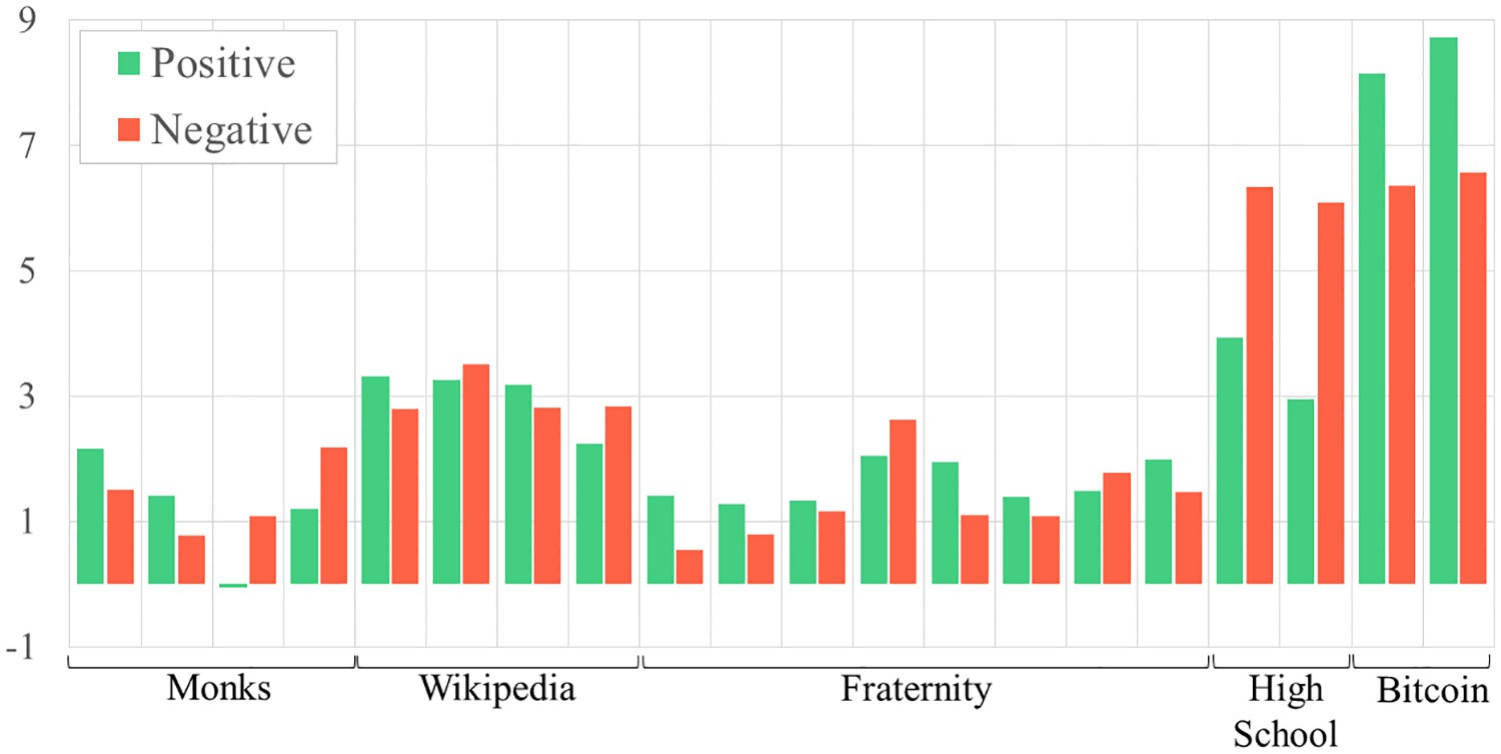
Reciprocity ERGM coefficient values for positive versus negative networks grouped by network type.

**Fig 3:**
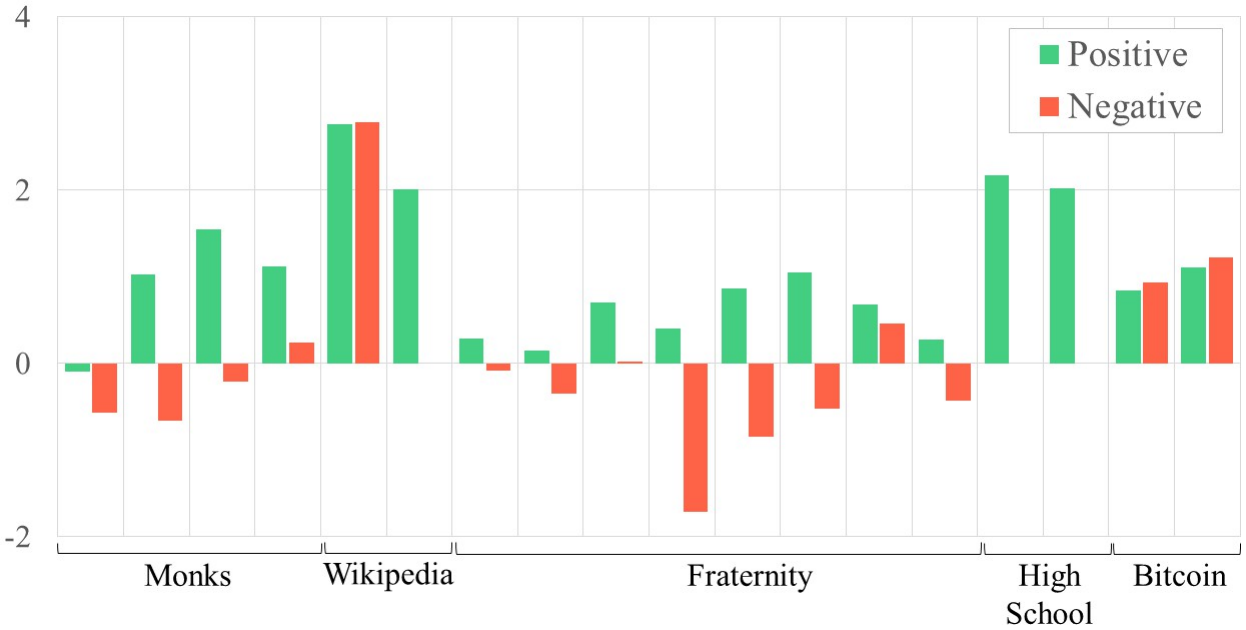
Transitivity ERGM coefficient values for positive versus negative networks grouped by network type.

**Fig 4:**
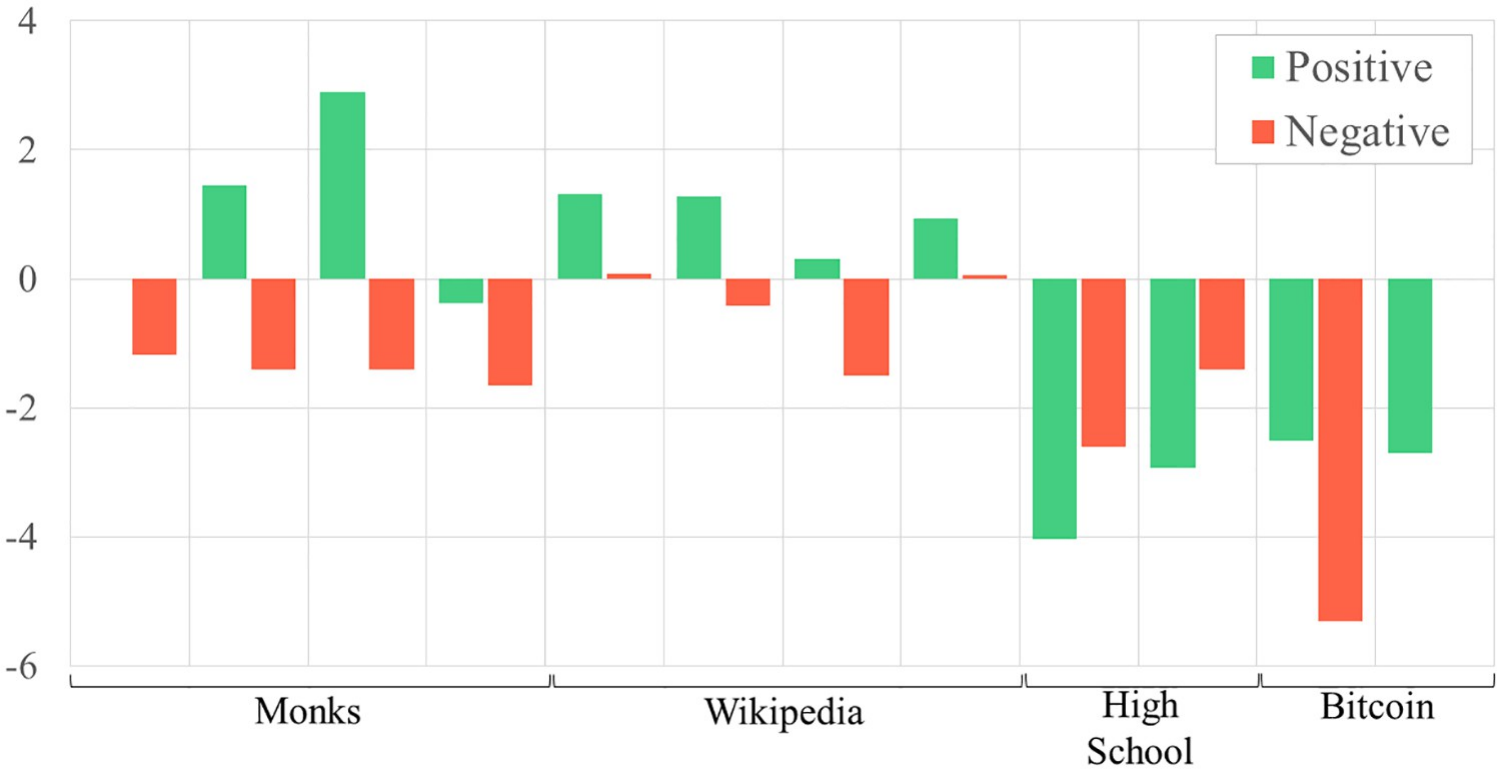
Outdegree skew ERGM coefficient values for positive versus negative networks grouped by network type.

When we considered the ERGM coefficients across each individual network, we also uncover some exceptions to the general trends discussed above (see Figs 2–4). For example, both cyberbullying networks exhibit higher, rather than similar, levels of reciprocity as the friendship networks that connect the same adolescents. In another case, many positive networks that consist of in-person interaction, such as the friendly bonds among adolescents and fraternity brothers, are defined by skewed in- and outdegree distributions. When positive interaction occurs online, as is the case when editing Wikipedia articles, graphs are characterized by more uniform in- and outdegree distributions.

### Structural signatures

By calculating the correlations between all sets of predicted probabilities, we found some evidence that unique structural signatures define positive and negative networks (Research Question 4). In many instances, the coefficients from ERGMs estimated on positive networks best predict the structural patterns of other positive networks (average correlation = 0.47), and ERGMs estimated on negative networks are better at predicting the patterns of other negative graphs (average correlation = 0.36) (see Table 2). When the coefficients of a positive network’s ERGM are used to predict the structure of a negative network, however, results are less precise (average correlation = 0.24). The same trend holds when the coefficients of a negative network’s ERGM are used to predict a positive network’s structure (average correlation = 0.22).

**Table 2:** Average correlation coefficient between networks’ predicted probabilities by genre and positive versus negative sentiment.

		**Positive**					**Negative**				
		Monks	Wiki	Frat	High Sch	Bitcoin	Monks	Wiki	Frat	High Sch	Bitcoin
**Positive**	Monks	0.557	0.535	0.701	0.656	0.555	0.220	0.510	0.014	0.497	0.545
	Wikipedia	0.222	0.398	-0.092	-0.106	-0.429	-0.379	0.062	-0.115	-0.383	-0.306
	Fraternity	0.734	0.534	0.887	0.627	0.617	0.538	0.480	0.423	0.678	0.624
	High Sch	0.616	0.207	0.709	0.888	0.478	0.295	0.371	-0.114	0.287	0.515
	Bitcoin	-0.097	-0.021	-0.096	0.026	0.202	0.023	0.179	0.041	0.022	0.169
**Negative**	Bitcoin	-0.097	-0.021	-0.096	0.026	0.202	0.023	0.179	0.041	0.022	0.169
	Monks	0.068	-0.021	0.488	0.203	0.192	0.767	0.219	0.548	0.621	0.301
	Wikipedia	0.065	-0.041	-0.429	0.220	0.357	-0.201	0.426	-0.399	-0.226	-0.096
	Fraternity	0.333	0.342	0.653	0.353	0.361	0.709	0.370	0.708	0.658	0.404
	High Sch	-0.275	-0.316	0.651	0.072	0.241	0.752	-0.386	0.704	0.891	0.536
	Bitcoin	-0.083	-0.176	-0.109	-0.079	-0.227	0.143	-0.049	0.044	0.378	0.403

In fact, for some of the networks in our sample, ERGMs of same-sign networks better predict the focal network’s structural patterns than the focal graph’s corresponding opposite-sign network. For example, the ERGM coefficients from networks of collaborative Wikipedia edits, amicable relations among fraternity brothers, adolescent friendships, and ratings of trust on a Bitcoin trading platform all better predict the structure of the monks’ positive influence network (average correlation = 0.74) than those of the monks’ negative influence network (correlation = -0.45). Note that the monks’ positive and negative influence networks include the same set of actors and are constructed from nearly identical survey items that were administered at the same point in time. With some exceptions (e.g., Wikipedia), most ERGM coefficients from negative networks tend to better predict the structure of a cyberbullying network (average correlation = 0.47) than those of the friendship network collected among the same actors (correlation = -0.50). These findings demonstrate that the sign of ties can play a more crucial role in determining the local patterns of networks than the type of actors or nature of the group.

## Discussion

Antagonistic, conflictual relationships characterize numerous forms of interpersonal behavior and carry particularly severe ramifications for social life. The structural features of these negative networks depart from more cooperative, pro-social graphs in important ways, and yet, until recently, these issues received little attention in the literature. A key objective of this study was to examine the extent to which small-scale, structural patterns can distinguish between comparable positive and negative social networks. According to meta-analyses of ERGMs estimated on 40 networks, the local structures that define positive and negative graphs are characterized by important differences, as well as notable similarities. Perhaps surprisingly, we found that both positive and negative networks are defined by higher levels of reciprocity than would be expected in equivalent random graphs. At the same time, local structural patterns tend to differ regarding triad and degree distribution patterns. Transitive patterns of triad closure are more likely to characterize connections in positive networks as compared to negative graphs, whereas skewed degree distributions define more negative than positive networks, on average. Findings reveal some trends towards structural signatures for negative and positive networks, while also pointing to important distinctions within our sample of graphs.

We found robust and consistent evidence for mutuality in networks defined by both friendly and antagonistic relations. After controlling for key structural factors and graph density, reciprocity patterns tend to be similar across our sample of positive and negative graphs in terms of both the large effect size and level of significance. Two actors are approximately 2.7 times more likely to be engaged in a mutual social connection than would be expected otherwise, regardless of whether links are defined by positive or negative valence. Mutuality in positive networks is not surprising and reflects the “norm of reciprocity,” in which people are expected to return supportive behaviors and sentiments [17]. Alternatively, the overrepresentation of reciprocated dyads in our negative datasets likely involves retaliation for confrontational behavior, such as the deletions of rival users’ posts on Wikipedia [16] or acts of revenge for online bullying [22]. Direct, person-to-person conflict within institutional settings also contributes to reciprocity in the antagonistic fraternity and monastery datasets, despite the possible underrepresentation of these connections due to social norms that discourage direct expressions of dislike.

Our reciprocity results diverge from some studies that find notably lower levels of mutuality in negative as compared to positive networks, such as in online game attacks [25] and antagonistic ties among Honduran villagers [29]. These variations may be the result of different study designs and methodological approaches. Descriptively, our datasets contain a lower proportion of reciprocated dyads in negative networks when compared to their corresponding positive graphs (average proportions are 0.20 and 0.31, respectively). Many of the negative networks in our sample—especially those defined by online interactions—are characterized by high levels of sparsity, which may explain the relatively low proportions of mutuality. When we control for graph density and other structural configurations in multivariate models, however, differences in reciprocity between positive and negative networks diminish.

Variations in the consequences associated with specific behaviors are also apt to shape our mutuality findings for the negative networks in our sample. We maintain that the costs and rewards associated with reciprocating an antagonistic interaction differ widely depending on the broader context. For example, reciprocity is particularly high in our online aggression networks, even though previous work argues that the power asymmetries of adolescent bullying can inhibit mutual targeting [54]. Repaying online peer aggression typically faces lower costs than returning the fists of a more powerful foe. At the same time, when reciprocity does manifest in face-to-face peer aggression, retaliation may seem less costly than the social price of ignoring egregious bullying incidents [23].

Negative ties also can reap rewards [28], and the anticipated benefits of negative reciprocity diverge widely, depending on the nature of the harmful interaction and situational context [23]. For instance, removing a contribution to Wikipedia of someone who deleted your own entry could increase one’s scholarly reputation in the eyes of the online community, or at least forestall posts that detract from one’s public profile. A propensity for reciprocity of antagonism suggests, too, that struggles in our data may occur among relative status equals, who are vying back and forth for status and social positions (e.g., [55, 56]), rather than in hierarchical confrontations (e.g., [57]).

Trends towards transitivity in our positive networks are also not unexpected since previous work documents the phenomenon in multiple friendly, cooperative interactions (e.g., [10, 35]). The underrepresentation of closed, transitive triads in our negative networks is intriguing, however, given the continued debate regarding transitivity in antagonistic settings (e.g., is the “enemy of my enemy is my enemy?”) The dearth of these configurations in negative graphs is consistent with balance theory predictions, which suggest that triads consisting of three negative ties should be unstable and underrepresented. The lack of transitivity in certain negative networks also could represent hierarchical patterns in which high ranking actors direct action only to those one rank below them, and allow their “minions” to interact among lower status individuals (e.g., Mafia networks, [58]). In addition, these patterns could be due to the volatility of these harmful links. The status hierarchies of negative networks are frequently more ephemeral than their positive counterparts since actors on the lower rungs hold particularly vested interests in fighting their way up the social ladder by dissolving these transitive structures [23].

Distributions of indegree and outdegree in our data also yield interesting findings. For instance, the actors in our sample of negative networks consistently exhibit significantly skewed patterns of sending and receiving ties. Most actors are connected to few negative ties, if any, perhaps due to social norms that discourage actors from participating in negative interactions [13, 29]. Only a small minority are responsible for sending large volumes of negative ties, and an even smaller minority are the targets of these attacks.

When aggregated, we find that positive networks tend to lack significant degree skew. This non-significance appears to follow from opposite patterns of skew in young people’s friendships versus online connections. For example, in five fraternity graphs (mostly from later time periods) and two adolescent friendship networks, we find evidence that significant indegree skew defines these networks. Indegree skew in friendship networks reflects common patterns of preferential attachment in popularity, a trend documented routinely in the extant literature (e.g., [13, 39]). A significant absence of indegree skew, however, arises in the two trust networks from the Bitcoin trading platform and three of the cooperative Wikipedia editing graphs (all but the smallest). This lack of skew suggests that positive interactions on these online venues may be more evenly distributed among actors than is typical in friendship popularity contests. The absence of skew also defines patterns of outgoing ties for three positive Wikipedia networks, highlighting the collaborative environment of agreeable editing on the venue.

Modest evidence surfaces of distinctive structural signatures for positive and negative networks. The sign of actors’ ties influences the structural patterns of their graphs, and notably, we found that some networks are more similar to same-sign graphs than to opposite-sign graphs that include the same actors situated in an identical context. Interestingly, there are also key exceptions. For example, the current study provides evidence that the predictive patterns for online interaction networks often depart from those of other graphs. Variations by the mode and context of interaction across our negative graphs highlights the need to elaborate more detailed typologies of negative interaction [1], particularly regarding the costs and benefits of engaging in harmful confrontations [23].

Although there are several strengths to the research presented here, there are also limitations. The approach we take should be applied in the future to compare many network types not examined here, and other structural factors need to be considered, as well. The inclusion of alternate controls, such as cyclical triads or various other closure patterns, could generate different estimates. Additionally, while our models include terms for reciprocity, transitivity, and skew simultaneously, future work should consider how these processes interact since previous work finds that certain local network patterns are related in some positive networks [59]. Finally, the networks analyzed in the current study were collected at discrete time points. It would be informative to study the evolution of network structure over time with longitudinal data and temporal ERGMs or stochastic actor-oriented models, although the typical lack of stability in negative ties makes longitudinal work challenging (e.g., [13, 60]).

In conclusion, the structural underpinnings of agonistic networks often differ from those of their more cooperative counterparts. The positive graphs in our sample are more likely to display tendencies towards transitivity, while the negative graphs produce heavily skewed in- and outdegree distributions. At the same time, key exceptions to these patterns arise. The local structures that define certain genres of online social behavior often differ from that of face-to-face interaction. These discrepancies may be related to the visibility of interactions, and to the costs and benefits associated with extending a positive or negative tie across different situations. Future explorations of these network contexts could benefit from a framework that considers further the potential consequences of tie formation, and their implications for intriguing, small-scale network configurations.

## Supporting information

S1 File Supplemental materials. (DOCX)

S2 File Network data. (XLSX)
