# Supplementary material for: Reciprocity, transitivity, and skew: Comparing local structure in 40 positive and negative social networks
Source: PLoS One. 2022 May 20;17(5):e0267886. doi: 10.1371/journal.pone.0267886 (PMC9122197; doi:10.1371/journal.pone.0267886)
Supplement: S1 File — (DOCX) [file pone.0267886.s001.docx]

**Reciprocity, Transitivity, and Skew: Comparing Local Structure in 40 Positive and Negative Social Networks**

*Supplemental Materials*

**Part A. Summary of ERGM Results**

Table S1. ERGM results for positive Sampson’s monastery networks

|  | Like | | Positive Influence | | Esteem | | Praise | |
| --- | --- | --- | --- | --- | --- | --- | --- | --- |
| Edges | -1.396 | ** | -1.914 | *** | -2.893 | *** | -2.485 | *** |
|  | (0.535) |  | (0.409) |  | (0.613) |  | (0.652) |  |
| Mutual | 2.161 | *** | 1.399 | ** | -0.049 |  | 1.191 | + |
|  | (0.502) |  | (0.458) |  | (0.556) |  | (0.614) |  |
| GWESP | -0.091 |  | 1.023 | *** | 1.548 | *** | 1.125 | *** |
|  | (0.208) |  | (0.271) |  | (0.332) |  | (0.323) |  |
| GWDSP | -0.151 |  | -0.395 | *** | -0.327 | ** | -0.262 | * |
|  | (0.147) |  | (0.089) |  | (0.111) |  | (0.131) |  |
| GWI | -0.570 |  | 0.328 |  | 5.210 | + | 1.018 |  |
|  | (0.942) |  | (0.818) |  | (2.932) |  | (0.868) |  |
| GWO |  |  | 1.453 |  | 2.897 | * | -0.382 |  |
|  |  |  | (1.025) |  | (1.218) |  | (0.802) |  |
| AIC | 424.2 |  | 424.2 |  | 424.2 |  | 424.2 |  |
| BIC | 266.2 |  | 240.5 |  | 245.0 |  | 207.3 |  |

*Notes:* *** *p* < 0.001, ** *p* < 0.01, * *p* < 0.05. Standard errors are in parentheticals.

Table S2. ERGM results for negative Sampson’s monastery networks

|  | Dislike | | Negative Influence | | Disesteem | | Blame | |
| --- | --- | --- | --- | --- | --- | --- | --- | --- |
| Edges | -0.735 | + | -0.633 |  | -0.495 |  | -0.674 | + |
|  | (0.441) |  | (0.419) |  | (0.375) |  | (0.383) |  |
| Mutual | 1.496 | ** | 0.772 |  | 1.074 | * | 2.177 | ** |
|  | (0.569) |  | (0.554) |  | (0.506) |  | (0.667) |  |
| GWESP | -0.564 | * | -0.663 |  | -0.210 |  | 0.242 |  |
|  | (0.285) |  | (0.307) |  | (0.226) |  | (0.273) |  |
| GWDSP | -0.018 |  | 0.002 |  | -0.104 |  | -0.501 | *** |
|  | (0.131) |  | (0.121) |  | (0.111) |  | (0.147) |  |
| GWI | -3.039 | *** | -2.759 | ** | -3.372 | *** |  |  |
|  | (0.771) |  | (0.811) |  | (0.854) |  |  |  |
| GWO | -1.187 |  | -1.402 |  | -1.409 |  | -1.658 | * |
|  | (0.718) |  | (0.850) |  | (0.879) |  | (0.787) |  |
| AIC | 424.2 |  | 424.2 |  | 424.2 |  | 424.2 |  |
| BIC | 237.8 |  | 256.7 |  | 273.4 |  | 217.4 |  |

*Notes:* *** *p* < 0.001, ** *p* < 0.01, * *p* < 0.05. Standard errors are in parentheticals.

Table S3. ERGM results for positive Wikipedia networks

|  | Topic 1 | | Topic 2 | | Topic 3 | | Topic 4 | |
| --- | --- | --- | --- | --- | --- | --- | --- | --- |
| Edges | -7.414 | *** | -4.581 | *** | -8.209 | *** | -6.395 | *** |
|  | (0.150) |  | (0.168) |  | (0.102) |  | (0.162) |  |
| Mutual | 3.316 | *** | 3.248 | *** | 3.184 | *** | 2.235 | *** |
|  | (0.348) |  | (0.336) |  | (0.339) |  | (0.472) |  |
| GWESP |  |  |  |  | 2.756 | *** | 2.009 | *** |
|  |  |  |  |  | (0.219) |  | (0.252) |  |
| GWDSP |  |  |  |  |  |  |  |  |
|  |  |  |  |  |  |  |  |  |
| GWI | 1.087 | *** | -0.632 | * | 0.746 | *** | 0.546 | * |
|  | (0.173) |  | (0.301) |  | (0.115) |  | (0.216) |  |
| GWO | 0.938 | *** | 0.314 |  | 1.272 | *** | 1.320 | *** |
|  | (0.172) |  | (0.307) |  | (0.122) |  | (0.229) |  |
| AIC | 6001 |  | 1452 |  | 14265 |  | 3243 |  |
| BIC | 6042 |  | 1481 |  | 14324 |  | 3289 |  |

*Notes:* *** *p* < 0.001, ** *p* < 0.01, * *p* < 0.05. Standard errors are in parentheticals.

Table S4. ERGM results for negative Wikipedia networks

|  | Topic 1 | | Topic 2 | | Topic 3 | | Topic 4 | |
| --- | --- | --- | --- | --- | --- | --- | --- | --- |
| Edges | -7.133 | *** | -4.345 | *** | -7.771 | *** | -6.836 | *** |
|  | (0.200) |  | (0.216) |  | (0.132) |  | (0.315) |  |
| Mutual | 3.711 | *** | 3.508 | *** | 2.804 | *** | 2.835 | *** |
|  | (0.434) |  | (0.419) |  | (0.552) |  | (0.699) |  |
| GWESP |  |  |  |  | 2.788 | *** |  |  |
|  |  |  |  |  | (0.304) |  |  |  |
| GWDSP |  |  |  |  |  |  |  |  |
|  |  |  |  |  |  |  |  |  |
| GWI | 0.323 |  | -0.195 |  | 0.645 | *** | 0.766 | * |
|  | (0.208) |  | (0.338) |  | (0.150) |  | (0.317) |  |
| GWO | 0.052 |  | -1.509 | *** | -0.426 | *** | 0.080 |  |
|  | (0.202) |  | (0.330) |  | (0.138) |  | (0.293) |  |
| AIC | 3952 |  | 1022 |  | 9824 |  | 1787 |  |
| BIC | 3994 |  | 1052 |  | 9884 |  | 1823 |  |

*Notes:* *** *p* < 0.001, ** *p* < 0.01, * *p* < 0.05. Standard errors are in parentheticals.

Table S5. ERGM results for positive Newcomb fraternity networks (part 1)

|  | Wave 0 | | Wave 1 | | Wave 2 | | Wave 3 | |
| --- | --- | --- | --- | --- | --- | --- | --- | --- |
| Edges | -1.402 | ** | -1.024 | * | -1.915 | *** | -0.009 |  |
|  | (0.465) |  | (0.449) |  | (0.505) |  | (0.564) |  |
| Mutual | 1.411 | ** | 1.279 | ** | 1.322 | ** | 2.049 | *** |
|  | (0.459) |  | (0.457) |  | (0.447) |  | (0.458) |  |
| GWESP | 0.288 |  | 0.151 |  | 0.709 | * | 0.403 |  |
|  | (0.266) |  | (0.258) |  | (0.282) |  | (0.250) |  |
| GWDSP | -0.104 |  | -0.098 |  | -0.186 |  | -0.334 | *** |
|  | (0.122) |  | (0.120) |  | (0.096) |  | (0.095) |  |
| GWI | -1.332 |  | -1.775 | * | -0.110 |  | -1.389 |  |
|  | (0.880) |  | (0.843) |  | (0.984) |  | (1.101) |  |
| GWO |  |  |  |  |  |  |  |  |
|  |  |  |  |  |  |  |  |  |
| AIC | 335.7 |  | 335.8 |  | 322.8 |  | 316.3 |  |
| BIC | 353.7 |  | 353.9 |  | 340.8 |  | 334.3 |  |

*Notes:* *** *p* < 0.001, ** *p* < 0.01, * *p* < 0.05. Standard errors are in parentheticals.

Table S6. ERGM results for positive Newcomb fraternity networks (part 2)

|  | Wave 4 | | Wave 5 | | Wave 6 | | Wave 7 | |
| --- | --- | --- | --- | --- | --- | --- | --- | --- |
| Edges | -0.582 |  | -1.153 | ** | -1.043 | * | -0.214 |  |
|  | (0.584) |  | (0.384) |  | (0.423) |  | (0.469) |  |
| Mutual | 1.944 | *** | 1.387 | *** | 1.472 | ** | 1.977 | *** |
|  | (0.478) |  | (0.331) |  | (0.562) |  | (0.495) |  |
| GWESP | 0.864 | * | 1.054 | ** | 0.678 | * | 0.275 |  |
|  | (0.360) |  | (0.344) |  | (0.269) |  | (0.156) |  |
| GWDSP | -0.327 | *** | -0.342 | *** | -0.299 | ** | -0.309 | *** |
|  | (0.086) |  | (0.067) |  | (0.113) |  | (0.090) |  |
| GWI | -2.212 | ** | -1.468 |  | -1.750 | * | -2.507 | ** |
|  | (0.786) |  | (0.795) |  | (0.793) |  | (0.927) |  |
| GWO |  |  |  |  |  |  |  |  |
|  |  |  |  |  |  |  |  |  |
| AIC | 304 |  | 309.2 |  | 390.9 |  | 313.6 |  |
| BIC | 322 |  | 327.3 |  | 408.9 |  | 331.7 |  |

*Notes:* *** *p* < 0.001, ** *p* < 0.01, * *p* < 0.05. Standard errors are in parentheticals.

Table S7. ERGM results for negative Newcomb fraternity networks (part 1)

|  | Wave 0 | | Wave 1 | | Wave 2 | | Wave 3 | |
| --- | --- | --- | --- | --- | --- | --- | --- | --- |
| Edges | -0.539 |  | -0.512 |  | -0.975 | * | -1.166 |  |
|  | (0.572) |  | (0.600) |  | (0.490) |  | (2.180) |  |
| Mutual | 0.536 |  | 0.793 | * | 1.147 | ** | 2.616 | * |
|  | (0.385) |  | (0.366) |  | (0.406) |  | (1.333) |  |
| GWESP | -0.082 |  | -0.352 |  | 0.025 |  | -1.717 |  |
|  | (0.336) |  | (0.346) |  | (0.287) |  | (2.055) |  |
| GWDSP | 0.061 |  | 0.277 |  | 0.048 |  | 3.009 | * |
|  | (0.169) |  | (0.225) |  | (0.150) |  | (1.474) |  |
| GWI | -5.401 | ** | -7.882 | ** | -5.168 | ** | -36.403 | * |
|  | (2.024) |  | (2.703) |  | (1.599) |  | (18.463) |  |
| GWO |  |  |  |  |  |  |  |  |
|  |  |  |  |  |  |  |  |  |
| AIC | 357.5 |  | 352.7 |  | 353.5 |  | 545.8 |  |
| BIC | 375.6 |  | 370.7 |  | 370.6 |  | 563.8 |  |

*Notes:* *** *p* < 0.001, ** *p* < 0.01, * *p* < 0.05. Standard errors are in parentheticals.

Table S8. ERGM results for negative Newcomb fraternity networks (part 2)

|  | Wave 4 | | Wave 5 | | Wave 6 | | Wave 7 | |
| --- | --- | --- | --- | --- | --- | --- | --- | --- |
| Edges | 0.193 |  | 1.125 | * | -0.660 |  | -0.227 |  |
|  | (0.464) |  | (0.520) |  | (0.390) |  | (0.396) |  |
| Mutual | 1.092 | ** | 1.071 | ** | 1.776 | ** | 1.453 | *** |
|  | (0.399) |  | (0.407) |  | (0.545) |  | (0.379) |  |
| GWESP | -0.847 | * | -0.521 | ** | 0.457 |  | -0.427 |  |
|  | (0.334) |  | (0.161) |  | (0.277) |  | (0.250) |  |
| GWDSP | 0.372 |  | 0.178 |  | -0.330 | ** | -0.036 |  |
|  | (0.270) |  | (0.155) |  | (0.107) |  | (0.124) |  |
| GWI | -8.024 | ** | -7.099 | *** | -3.000 | ** | -5.621 | ** |
|  | (2.536) |  | (1.472) |  | (0.913) |  | (2.109) |  |
| GWO |  |  |  |  |  |  |  |  |
|  |  |  |  |  |  |  |  |  |
| AIC | 348.2 |  | 319.1 |  | 434.3 |  | 342.1 |  |
| BIC | 366.2 |  | 337.1 |  | 452.4 |  | 360.2 |  |

*Notes:* *** *p* < 0.001, ** *p* < 0.01, * *p* < 0.05. Standard errors are in parentheticals.

Table S9. ERGM results for positive and negative high school networks

|  | Friendship | | | | Cyber Aggression | | | |
| --- | --- | --- | --- | --- | --- | --- | --- | --- |
|  | Time 1 | | Time 2 | | Time 1 | | Time 2 | |
| Edges | -4.434 | *** | -4.442 | *** | -4.278 | *** | -5.202 | *** |
|  | (0.043) |  | (0.039) |  | (0.422) |  | (0.348) |  |
| Mutual | 3.919 | *** | 2.665 | *** | 6.328 | *** | 6.084 | *** |
|  | (0.081) |  | (0.065) |  | (0.890) |  | (0.703) |  |
| GWESP | 2.177 | *** | 2.158 | *** |  |  |  |  |
|  | (0.034) |  | (0.028) |  |  |  |  |  |
| GWDSP | -0.191 | *** | -0.171 | *** |  |  |  |  |
|  | (0.005) |  | (0.004) |  |  |  |  |  |
| GWI | -1.362 | *** | 0.323 |  | -4.084 | *** | -3.698 | *** |
|  | (0.249) |  | (0.238) |  | (0.432) |  | (0.322) |  |
| GWO | -4.034 | *** |  |  | -2.607 | *** | -1.411 | *** |
|  | (0.208) |  |  |  | (0.497) |  | (0.396) |  |
| AIC | 41797 |  | 48269 |  | 745.8 |  | 1368 |  |
| BIC | 41865 |  | 48326 |  | 791.1 |  | 1413 |  |

*Notes:* *** *p* < 0.001, ** *p* < 0.01, * *p* < 0.05. Standard errors are in parentheticals.

Table S10. ERGM results for positive and negative Bitcoin OTC trust networks

|  | Trust | | | | Distrust | | | |
| --- | --- | --- | --- | --- | --- | --- | --- | --- |
|  | Time 1 | | Time 2 | | Time 1 | | Time 2 | |
| Edges | -7.418 | *** | -7.923 | *** | -6.367 | *** | -6.760 | *** |
|  | (0.034) |  | (0.028) |  | (0.037) |  | (0.027) |  |
| Mutual | 8.145 | *** | 8.716 | *** | 6.355 | *** | 6.570 | *** |
|  | (0.076) |  | (0.062) |  | (0.236) |  | (0.144) |  |
| GWESP | 0.846 | *** | 1.110 | *** | 0.941 | *** | 1.220 | *** |
|  | (0.024) |  | (0.035) |  | (0.136) |  | (0.136) |  |
| GWDSP |  |  |  |  |  |  |  |  |
|  |  |  |  |  |  |  |  |  |
| GWI | 0.303 | ** | 0.269 | *** |  |  | -4.171 | *** |
|  | (0.094) |  | (0.078) |  |  |  | (0.085) |  |
| GWO | -2.515 | *** | -2.701 | *** | -5.305 | *** |  |  |
|  | (0.102) |  | (0.107) |  | (0.145) |  |  |  |
| AIC | 95429 |  | 134538 |  | 12775 |  | 29900 |  |
| BIC | 95495 |  | 134607 |  | 12827 |  | 29956 |  |

*Notes:* *** *p* < 0.001, ** *p* < 0.01, * *p* < 0.05. Standard errors are in parentheticals.

**Part B. Summary of ERGM Decay Parameters**

We include four ERGM terms in our models utilize decay parameters: geometrically weighted edge-wise shared partner (GWESP), geometrically weighted dyad-wise shared partner (GWDSP), geometrically weighted outdegree distribution (GWI), and geometrically weighted indegree distribution (GWO). A summary of the values assigned to the decay parameters for each specific ERGM term is presented below (see Table S11).

Table S11. Summary of ERGM decay parameters by network type

|  | GWESP | GWDSP | GWISP | GWO |
| --- | --- | --- | --- | --- |
| *Sampson’s Monastery* |  |  |  |  |
| Like | 0.25 | 0.25 | 0.25 |  |
| Positive Influence | 0.25 | 0.25 | 0.25 | 0.1 |
| Esteem | 0.25 | 0.25 | 0.25 | 0.25 |
| Praise | 0.25 | 0.25 | 0.25 | 0.25 |
| Dislike | 0.25 | 0.25 | 0.25 | 0.5 |
| Negative Influence | 0.25 | 0.25 | 0.25 | 0.25 |
| Disesteem | 0.25 | 0.25 | 0.25 | 0.25 |
| Blame | 0.25 | 0.25 | 0.25 |  |
| *Wikipedia* |  |  |  |  |
| Topic 1: Collaborative Edits |  |  | 0.1 | 0.1 |
| Topic 2: Collaborative Edits |  |  | 0.1 | 0.1 |
| Topic 3: Collaborative Edits | 0.1 |  | 0.1 | 0.1 |
| Topic 4: Collaborative Edits | 0.25 |  | 0.1 | 0.1 |
| Topic 1: Deletions |  |  | 0.1 | 0.1 |
| Topic 2: Deletions |  |  | 0.1 | 0.1 |
| Topic 3: Deletions | 2 |  | 0.1 | 0.1 |
| Topic 4: Deletions |  |  | 0.1 | 0.1 |
| *Newcomb’s Fraternity* |  |  |  |  |
| Wave 0: Top 5 | 0.1 | 0.1 | 0.5 |  |
| Wave 1: Top 5 | 0.1 | 0.1 | 0.75 |  |
| Wave 2: Top 5 | 0.25 | 0.25 | 0.25 |  |
| Wave 3: Top 5 | 0.1 | 1.25 | 0.5 |  |
| Wave 4: Top 5 | 0.1 | 1.25 | 0.5 |  |
| Wave 5: Top 5 | 0.1 | 0.75 | 0.25 |  |
| Wave 6: Top 5 | 0.25 | 0.25 | 0.5 |  |
| Wave 7: Top 5 | 0.75 | 1.25 | 0.25 |  |
| Wave 0: Bottom 5 | 0.25 | 0.25 | 0.25 |  |
| Wave 1: Bottom 5 | 0.25 | 0.25 | 0.25 |  |
| Wave 2: Bottom 5 | 0.25 | 0.25 | 0.25 |  |
| Wave 3: Bottom 5 | 0.1 | 0.1 | 0.25 |  |

Table S11 Continued. Summary of ERGM decay parameters by network type

|  | GWESP | GWDSP | GWI | GWO |
| --- | --- | --- | --- | --- |
| *Newcomb’s Fraternity Cont.* |  |  |  |  |
| Wave 4: Bottom 5 | 0.1 | 0.1 | 0.5 |  |
| Wave 5: Bottom 5 | 0.1 | 0.1 | 0.1 |  |
| Wave 6: Bottom 5 | 0.1 | 0.25 | 0.1 |  |
| Wave 7: Bottom 5 | 0.1 | 0.25 | 0.1 |  |
| *High School* |  |  |  |  |
| Time 1: Friendship | 0.1 | 0.5 | 0.1 | 0.1 |
| Time 2: Friendship | 0.25 | 0.25 | 0.25 |  |
| Time 1: Cyber Aggression |  |  | 0.1 | 0.1 |
| Time 2: Cyber Aggression |  |  | 0.1 | 0.1 |
| *Bitcoin OTC* |  |  |  |  |
| Time 1: Trust | 0.25 |  | 0.25 | 0.5 |
| Time 2: Trust | 0.25 |  | 0.1 | 0.1 |
| Time 1: Distrust | 0.5 |  |  | 0.1 |
| Time 2: Distrust | 0.25 |  | 0.25 |  |

*Note:* Missing values indicate that the term was not included.

**Part C. Goodness of Fit and Diagnostic Tests**

We conduct a variety of convergence diagnostics, calculate goodness of fit statistics, and assess the risk of collinearity for all ERGMs presented in the manuscript. Following Hunter et al. (2008), we assess convergence and goodness of fit by comparing the observed network’s (1) indegree distribution, (2) outdegree distribution, (3) minimum geodesic distance distribution, (4) edgewise shared partner distribution, and (5) triad census with those of networks generated from the ERGMs. We also consider the correlations between the variables included in each ERGM, as well as all parameters’ Variation Inflation Factors (VIFs) to assess the risk of multicollinearity in the models (Duxbury 2018).

Results from diagnostic tests are presented below for a positive and negative ERGM from each of the five genres (see Tables S12 through S31). Given that our models include only basic, structural variables, and do not have any individual- or dyad-level variables, the ERGMs we estimate fit our observed networks relatively well. Yet there are some exceptions. For instance, the ERGM estimated on the Bitcoin OTC trust network from Time 1 can overestimate actors’ reachability, as the minimum geodesic distance between actor pairs tends to be less than in the observed network. Across all networks, there are some parameters that are highly correlated. However, VIFs suggest that the ERGMs are not at high risk of multicollinearity.

Table S12. Correlation matrix and VIFs for the positive influence network from Sampson’s monastery study

|  | **Correlations** | | | | | |  | **VIFs** |
| --- | --- | --- | --- | --- | --- | --- | --- | --- |
|  | Edges | Mutual | GWESP | GWDSP | GWI | GWO |  |  |
| Edges | 1.000 |  |  |  |  |  |  |  |
| Mutual | 0.955 | 1.000 |  |  |  |  |  | 1.455 |
| GWESP | 0.990 | 0.966 | 1.000 |  |  |  |  | 1.626 |
| GWDSP | 0.981 | 0.935 | 0.972 | 1.000 |  |  |  | 1.068 |
| GWI | 0.552 | 0.545 | 0.514 | 0.571 | 1.000 |  |  | 1.217 |
| GWO | 0.381 | 0.374 | 0.347 | 0.393 | 0.368 | 1.000 |  | 1.117 |

Table S13. Goodness of fit summary for the positive influence network from Sampson’s monastery study

| 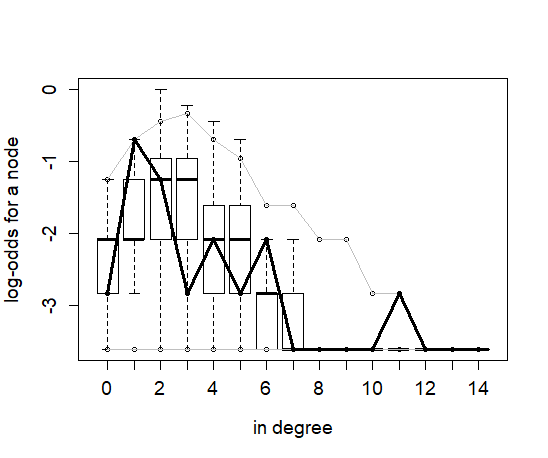 | 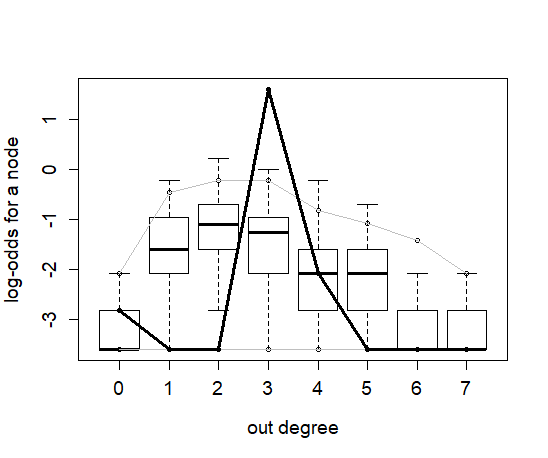 |
| --- | --- |
| 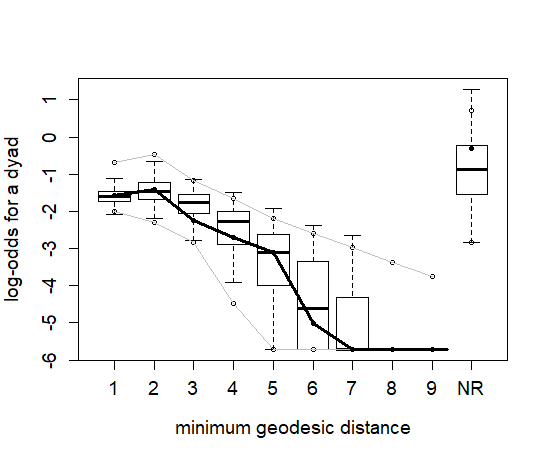 | 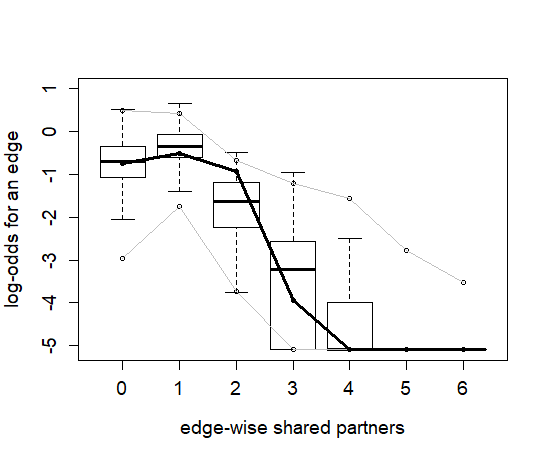 |
| 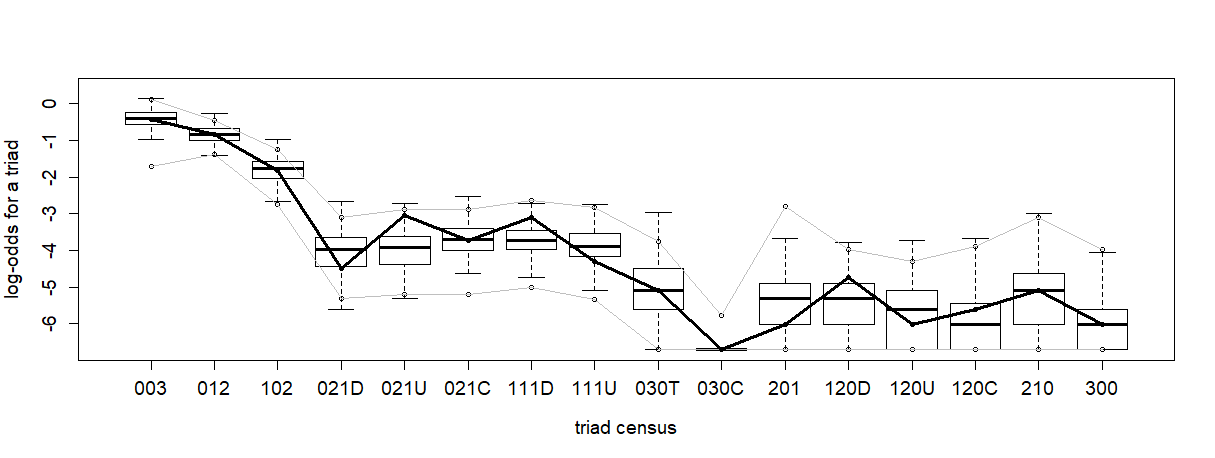 | |

Table S14. Correlation matrix and VIFs for the negative influence network from Sampson’s monastery study

|  | **Correlations** | | | | | |  | **VIFs** |
| --- | --- | --- | --- | --- | --- | --- | --- | --- |
|  | Edges | Mutual | GWESP | GWDSP | GWI | GWO |  |  |
| Edges | 1.000 |  |  |  |  |  |  |  |
| Mutual | 0.797 | 1.000 |  |  |  |  |  | 1.283 |
| GWESP | 0.747 | 0.495 | 1.000 |  |  |  |  | 1.564 |
| GWDSP | 0.958 | 0.813 | 0.767 | 1.000 |  |  |  | 1.444 |
| GWI | 0.861 | 0.709 | 0.552 | 0.851 | 1.000 |  |  | 1.202 |
| GWO | 0.837 | 0.669 | 0.541 | 0.814 | 0.720 | 1.000 |  | 1.118 |

Table S15. Goodness of fit summary for the negative influence network from Sampson’s monastery study

| 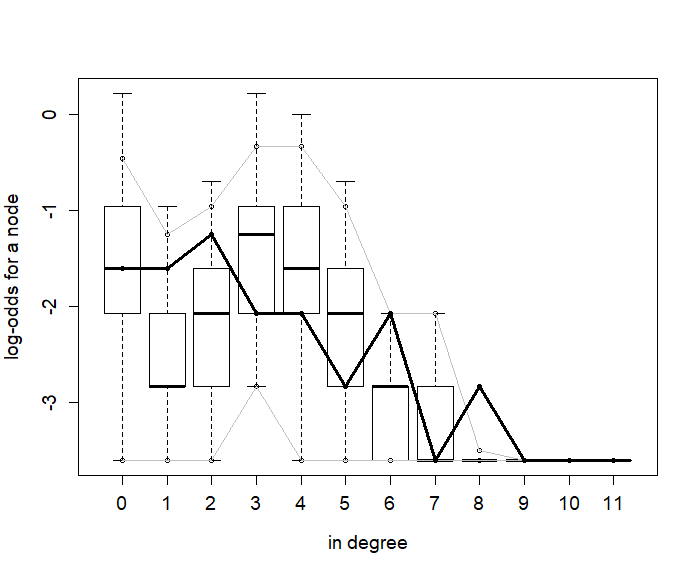 | 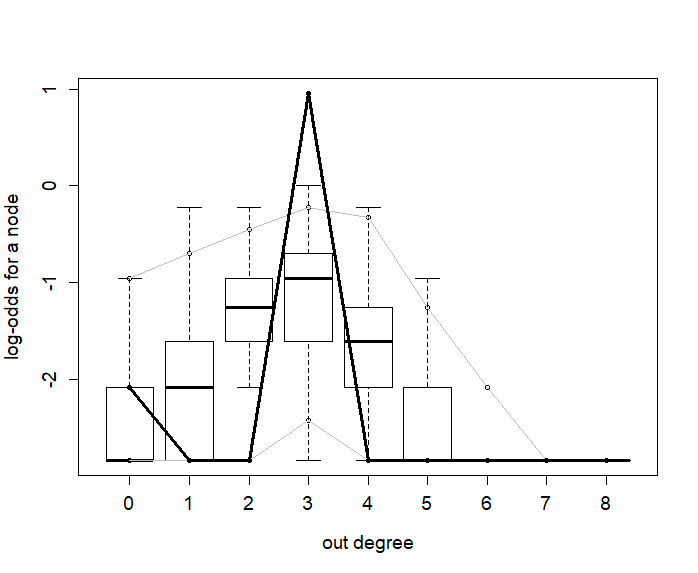 |
| --- | --- |
| 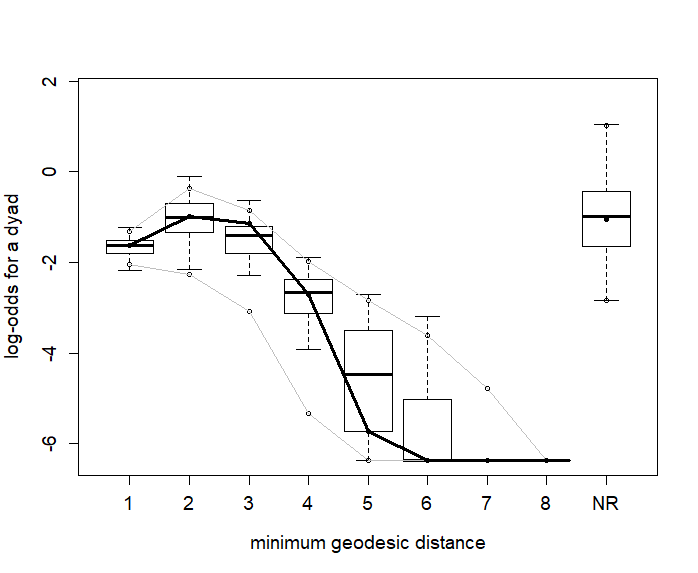 | 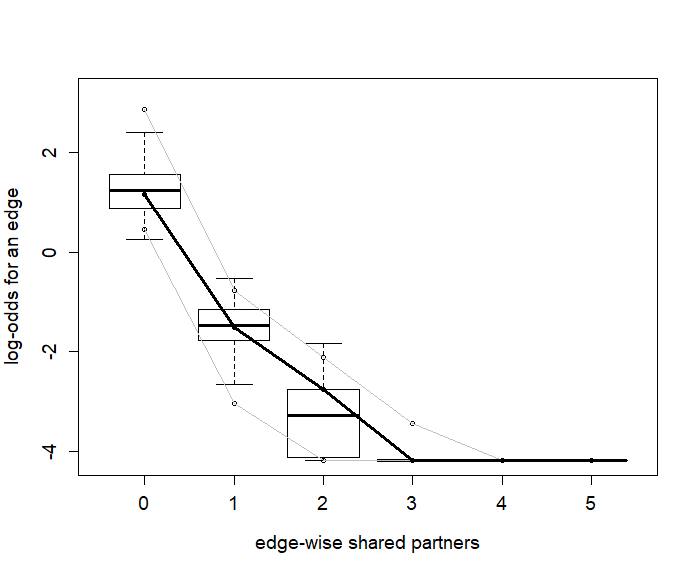 |
| 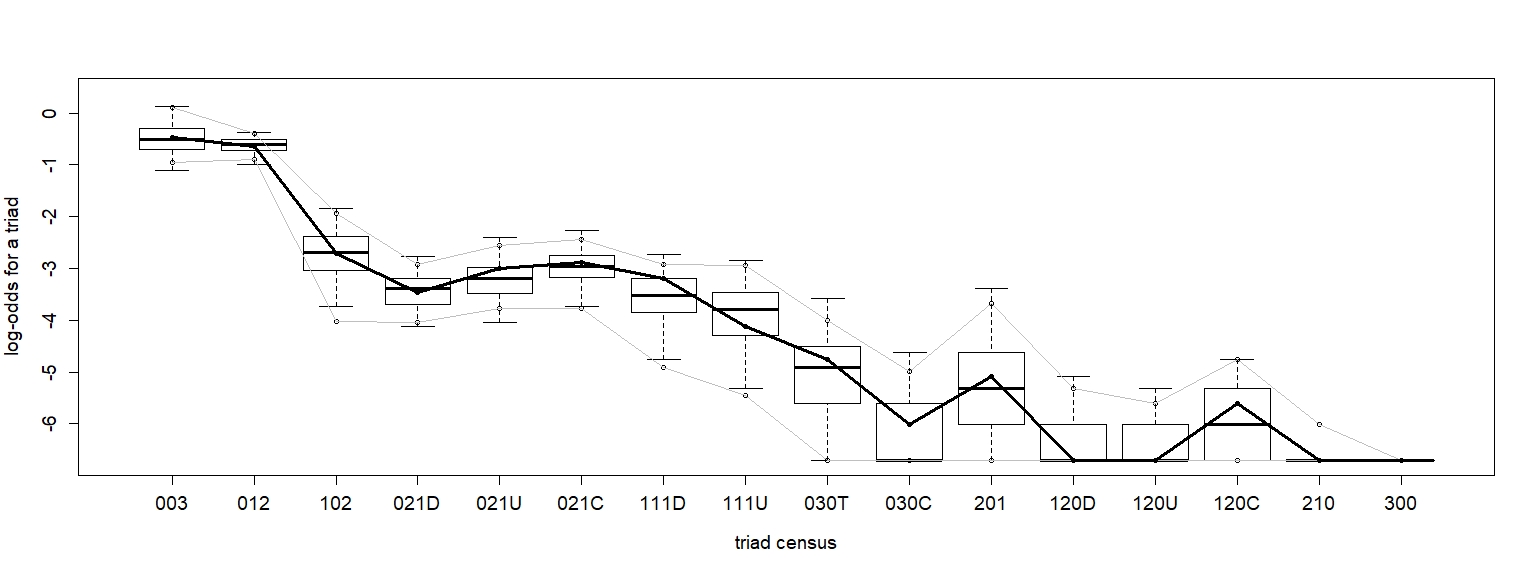 | |

Table S16. Correlation matrix and VIFs for the network of collaborative edits on the third Wikipedia topic

|  | **Correlations** | | | | |  | **VIFs** |
| --- | --- | --- | --- | --- | --- | --- | --- |
|  | Edges | Mutual | GWESP | GWI | GWO |  |  |
| Edges | 1.000 |  |  |  |  |  |  |
| Mutual | 0.132 | 1.000 |  |  |  |  | 1.455 |
| GWESP | 0.220 | 0.296 | 1.000 |  |  |  | 1.626 |
| GWI | 0.786 | 0.069 | 0.047 | 1.000 |  |  | 1.068 |
| GWO | 0.824 | 0.083 | 0.097 | 0.652 | 1.000 |  | 1.217 |

Table S17. Goodness of fit summary for the network of collaborative edits on the third Wikipedia topic

| 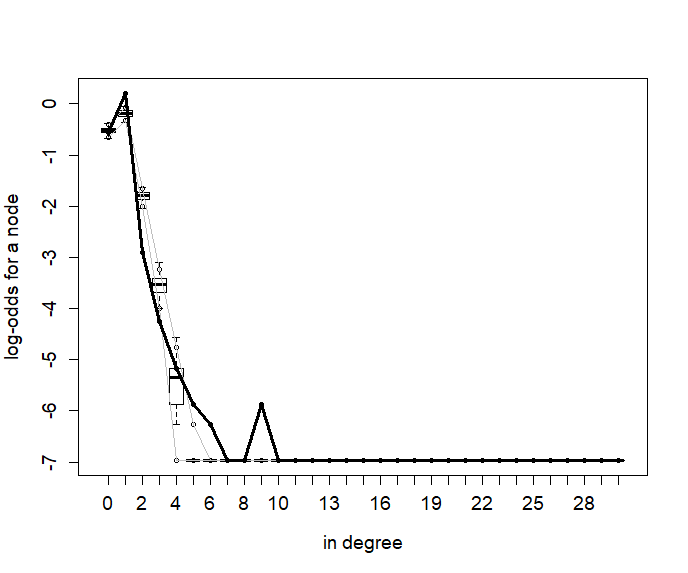 | 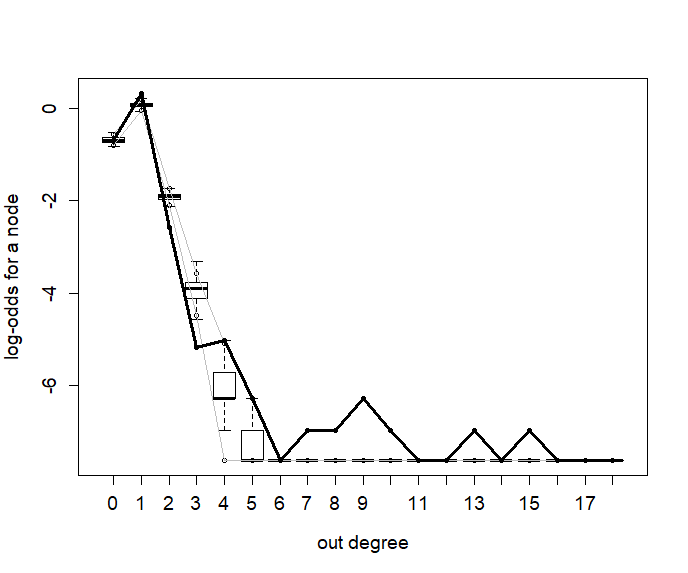 |
| --- | --- |
| 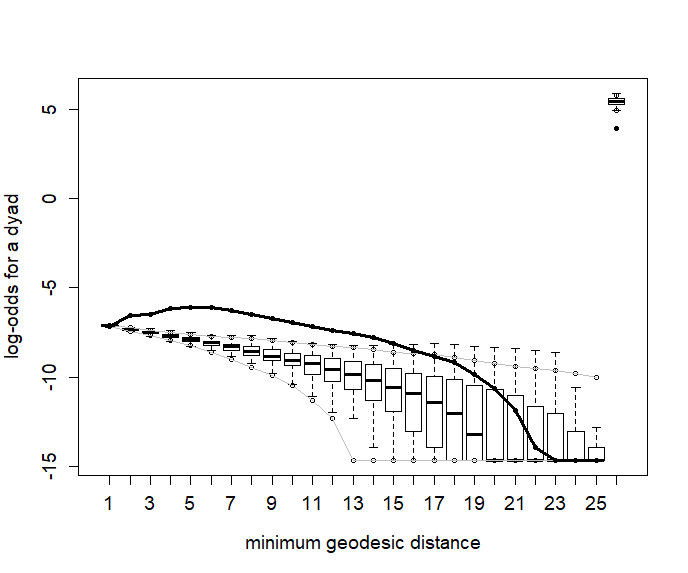 | 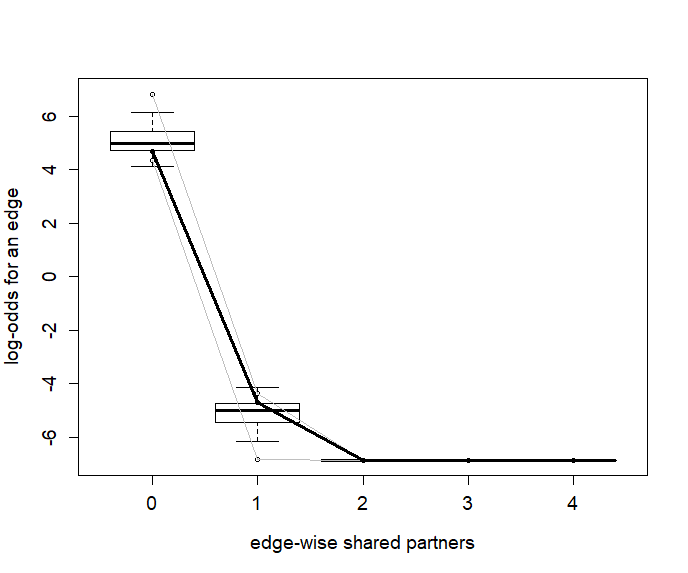 |
| 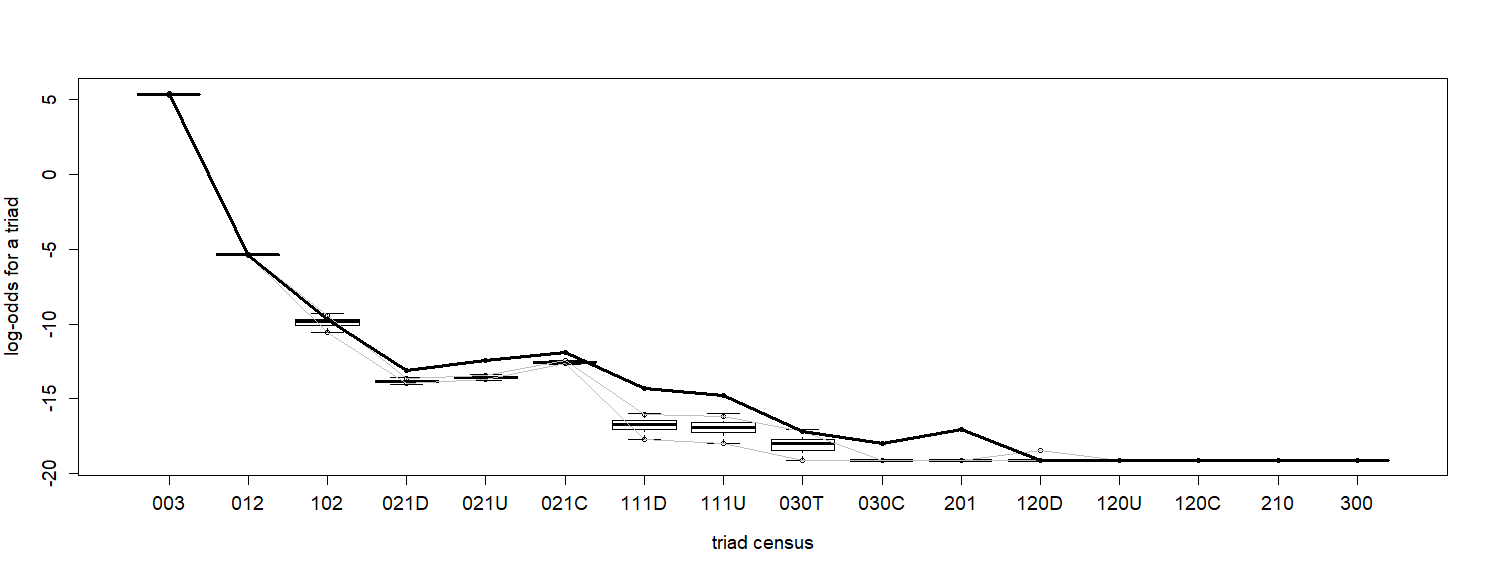 | |

Table S18. Correlation matrix and VIFs for the network of disagreeable edits on the third Wikipedia topic

|  | **Correlations** | | | | |  | **VIFs** |
| --- | --- | --- | --- | --- | --- | --- | --- |
|  | Edges | Mutual | GWESP | GWI | GWO |  |  |
| Edges | 1.000 |  |  |  |  |  |  |
| Mutual | 0.152 | 1.000 |  |  |  |  | 1.230 |
| GWESP | 0.203 | 0.449 | 1.000 |  |  |  | 1.333 |
| GWI | 0.919 | 0.105 | 0.086 | 1.000 |  |  | 1.074 |
| GWO | 0.855 | 0.098 | 0.097 | 0.797 | 1.000 |  | 1.024 |

Table S19. Goodness of fit summary for the network of disagreeable edits on the third Wikipedia topic

| 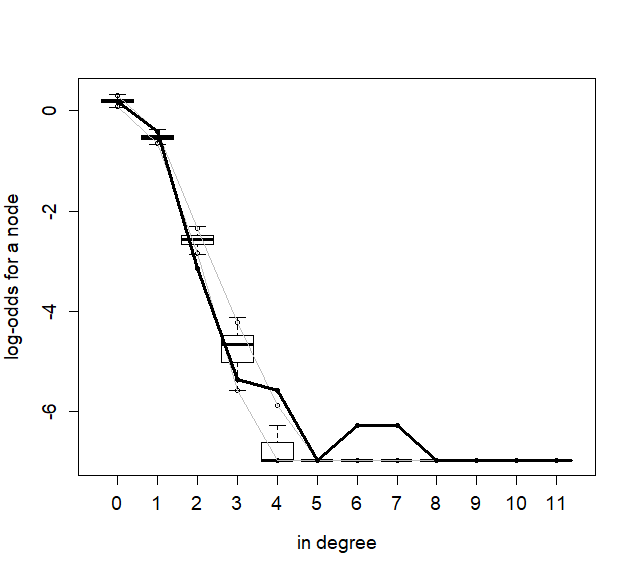 | 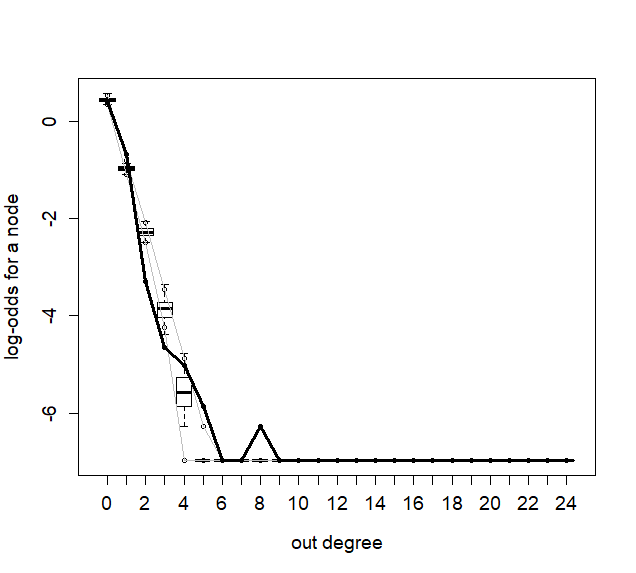 |
| --- | --- |
| 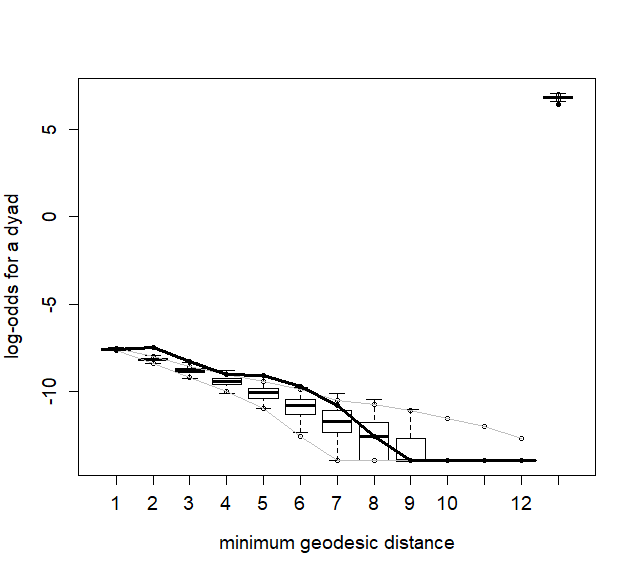 | 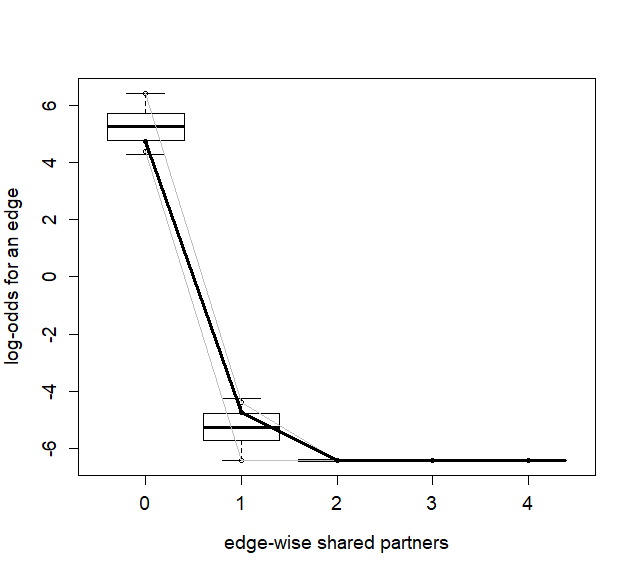 |
| 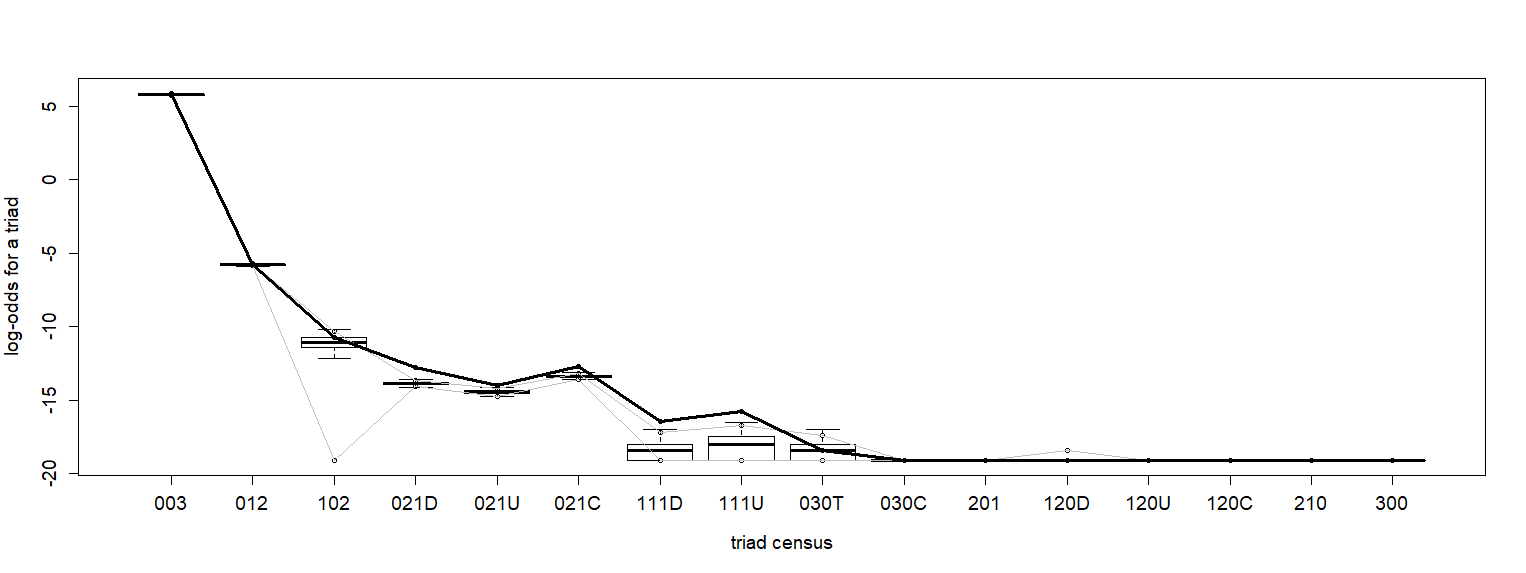 | |

Table S20. Correlation matrix and VIFs for the network of each actor’s top 5 nominations during the second wave of Newcomb’s fraternity study

|  | **Correlations** | | | | |  | **VIFs** |
| --- | --- | --- | --- | --- | --- | --- | --- |
|  | Edges | Mutual | GWESP | GWDSP | GWI |  |  |
| Edges | 1.000 |  |  |  |  |  |  |
| Mutual | 0.954 | 1.000 |  |  |  |  | 1.199 |
| GWESP | 0.990 | 0.961 | 1.000 |  |  |  | 1.691 |
| GWDSP | 0.987 | 0.943 | 0.976 | 1.000 |  |  | 1.074 |
| GWI | 0.755 | 0.700 | 0.696 | 0.772 | 1.000 |  | 1.543 |

Table S21. Goodness of fit summary for the network of each actor’s top 5 nominations during the second wave of Newcomb’s fraternity study

| 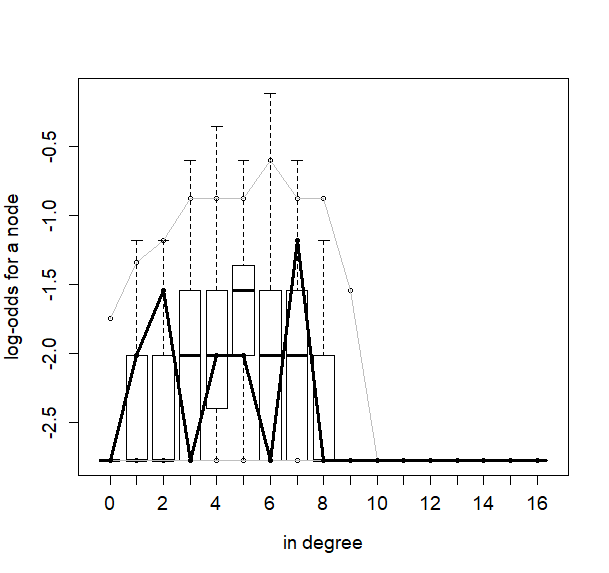 | 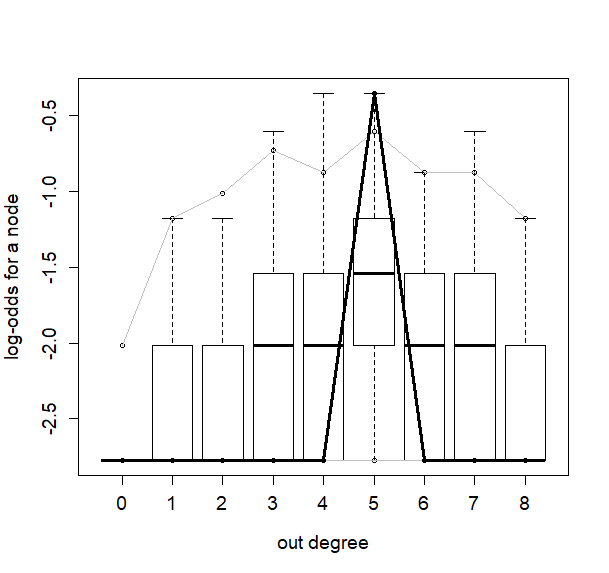 |
| --- | --- |
| 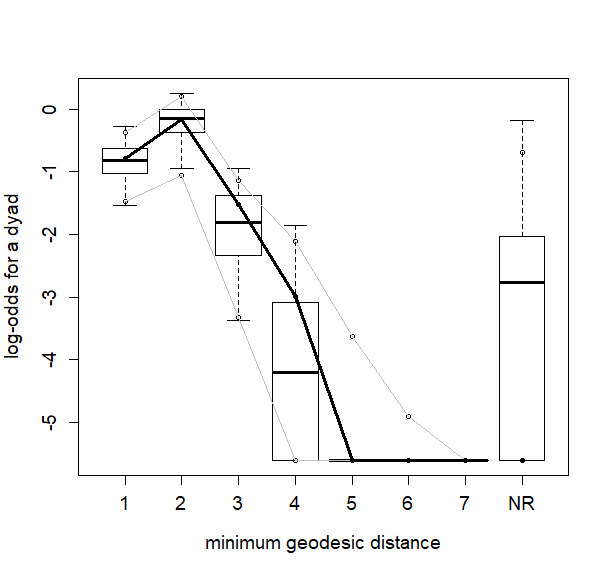 | 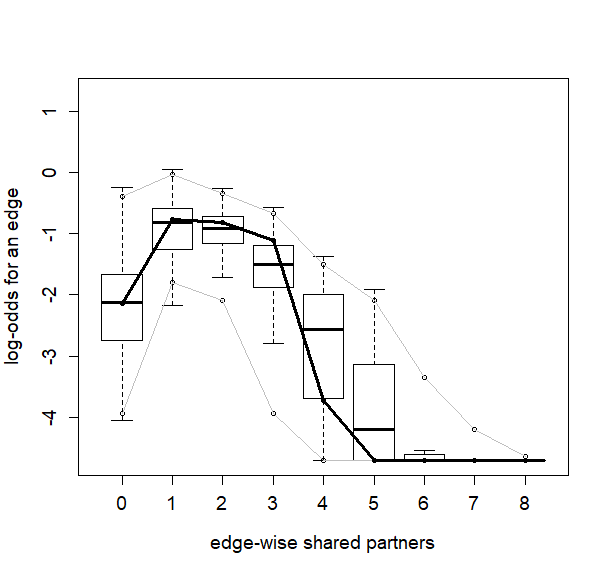 |
| 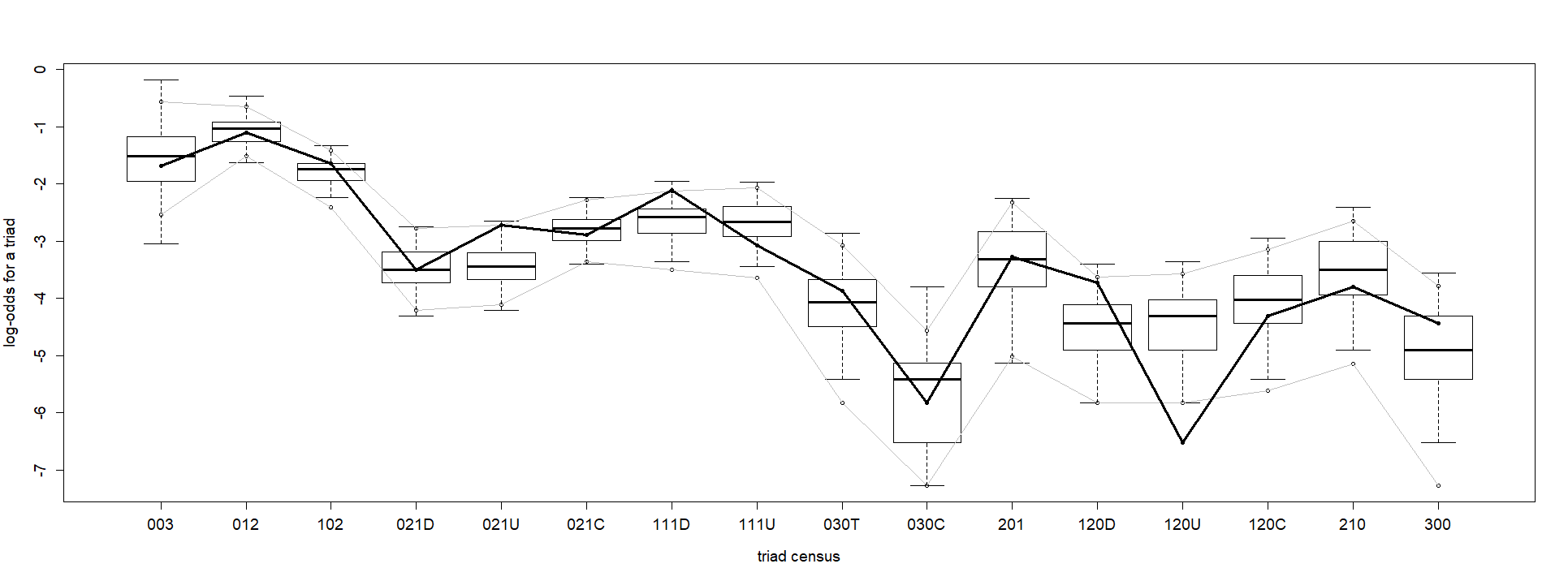 | |

Table S22. Correlation matrix and VIFs for the network of each actor’s bottom 5 nominations during the second wave of Newcomb’s fraternity study

|  | **Correlations** | | | | |  | **VIFs** |
| --- | --- | --- | --- | --- | --- | --- | --- |
|  | Edges | Mutual | GWESP | GWDSP | GWI |  |  |
| Edges | 1.000 |  |  |  |  |  |  |
| Mutual | 0.940 | 1.000 |  |  |  |  | 1.100 |
| GWESP | 0.983 | 0.940 | 1.000 |  |  |  | 2.360 |
| GWDSP | 0.987 | 0.934 | 0.966 | 1.000 |  |  | 2.315 |
| GWI | 0.928 | 0.863 | 0.868 | 0.955 | 1.000 |  | 3.888 |

Table S23. Goodness of fit summary for the network of each actor’s bottom 5 nominations during the second wave of Newcomb’s fraternity study

| 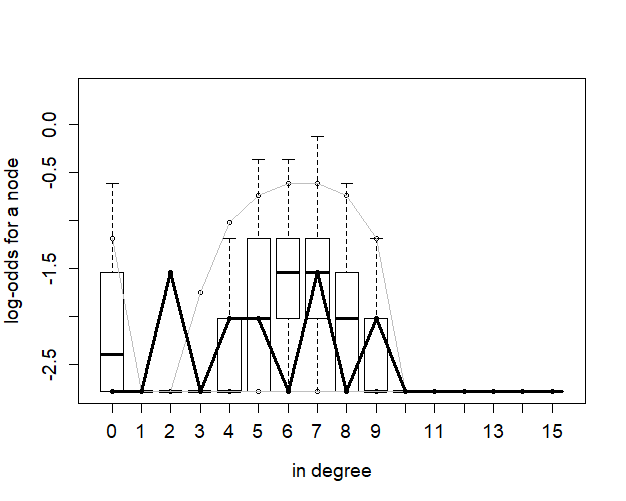 | 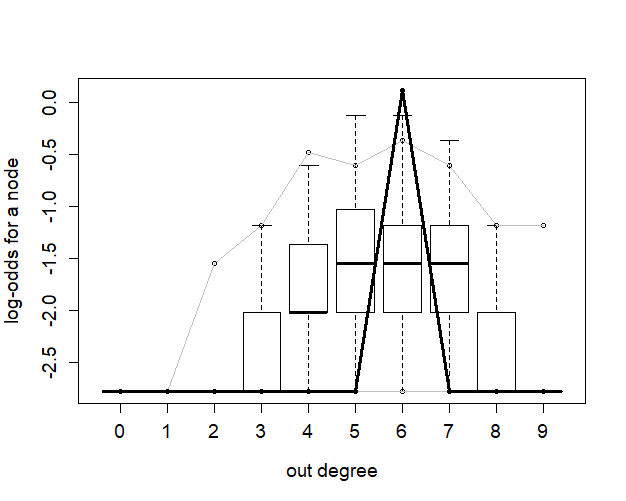 |
| --- | --- |
| 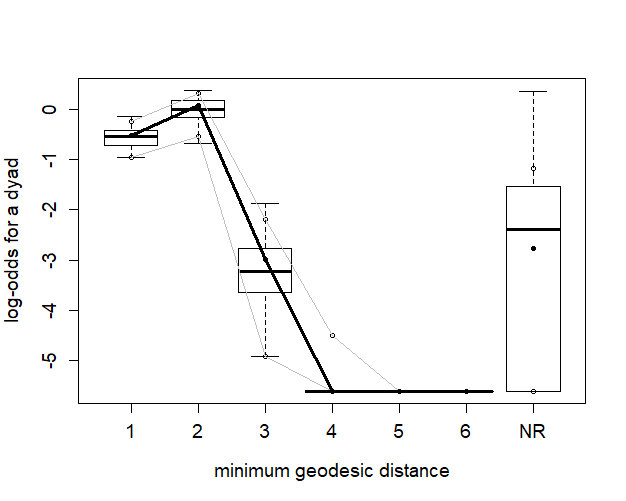 | 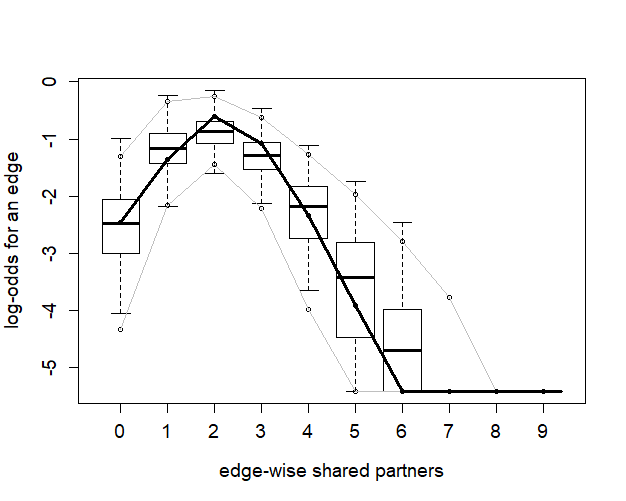 |
| 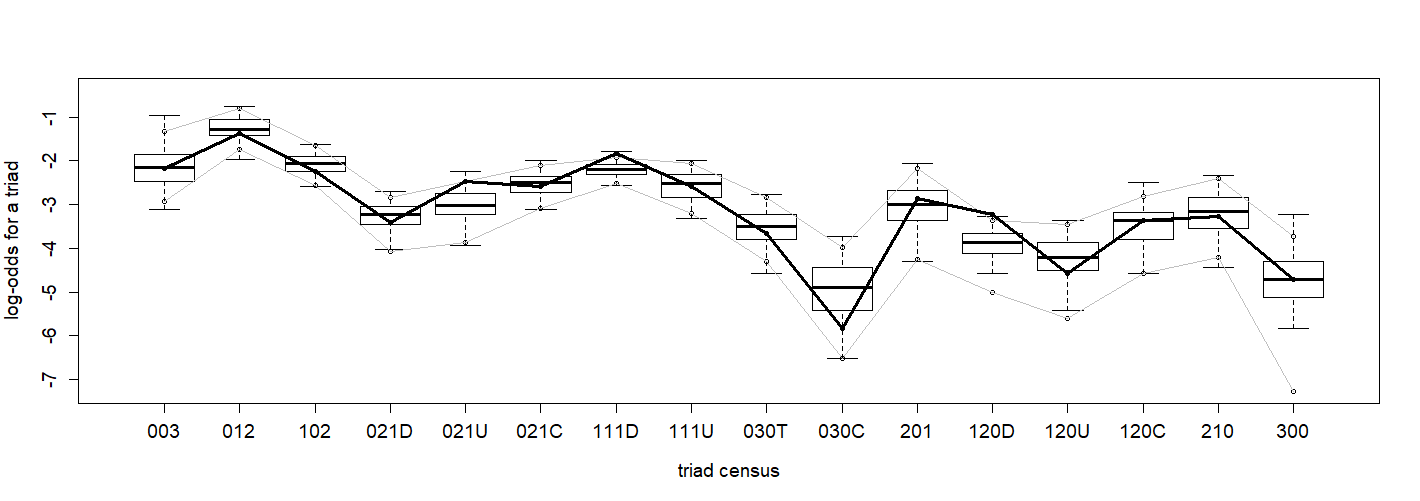 | |

Table S24. Correlation matrix and VIFs for the Time 1 friendship network from the Long Island Study

|  | **Correlations** | | | | | |  | **VIFs** |
| --- | --- | --- | --- | --- | --- | --- | --- | --- |
|  | Edges | Mutual | GWESP | GWDSP | GWI | GWO |  |  |
| Edges | 1.000 |  |  |  |  |  |  |  |
| Mutual | 0.660 | 1.000 |  |  |  |  |  | 3.653 |
| GWESP | 0.897 | 0.811 | 1.000 |  |  |  |  | 1.869 |
| GWDSP | 0.900 | 0.816 | 0.893 | 1.000 |  |  |  | 1.886 |
| GWI | 0.404 | 0.263 | 0.398 | 0.373 | 1.000 |  |  | 1.168 |
| GWO | 0.562 | 0.703 | 0.580 | 0.607 | -0.017 | 1.000 |  | 1.732 |

Table S25. Goodness of fit summary for the Time 1 friendship network from the Long Island Study

| 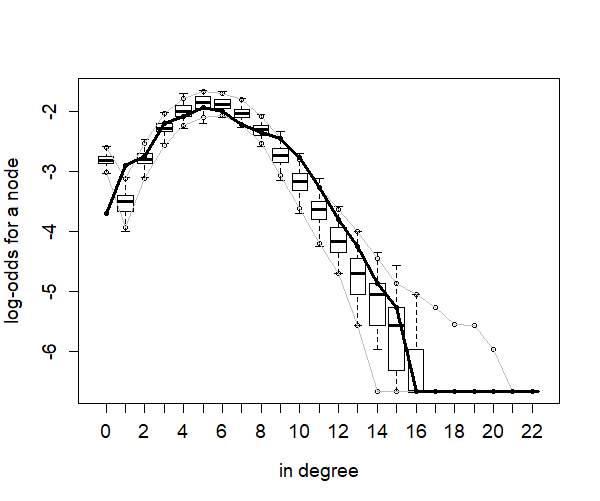 | 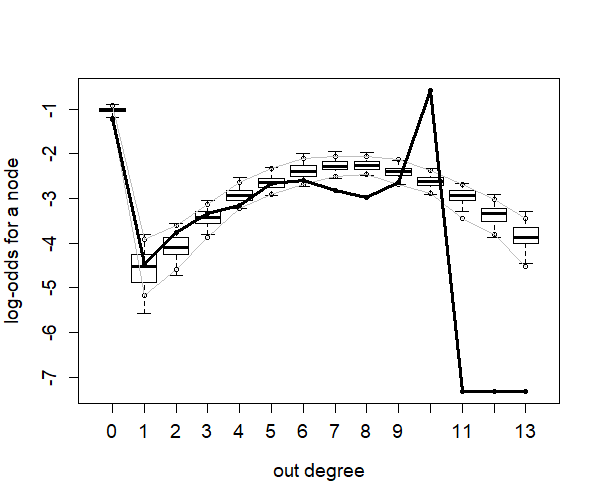 |
| --- | --- |
| 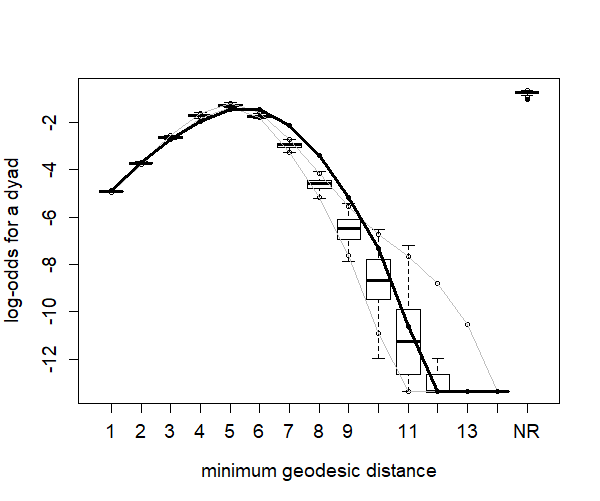 | 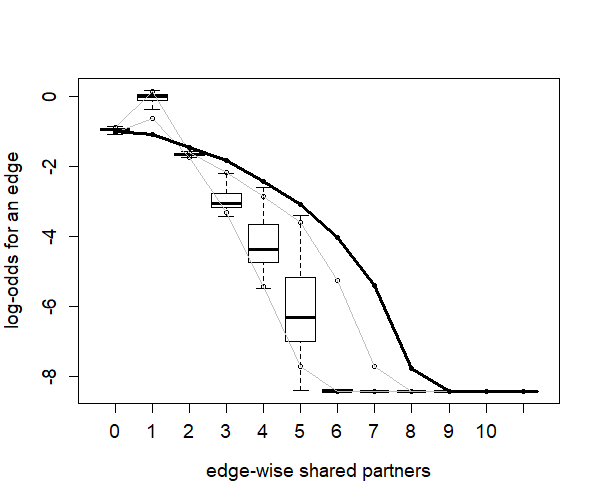 |
| 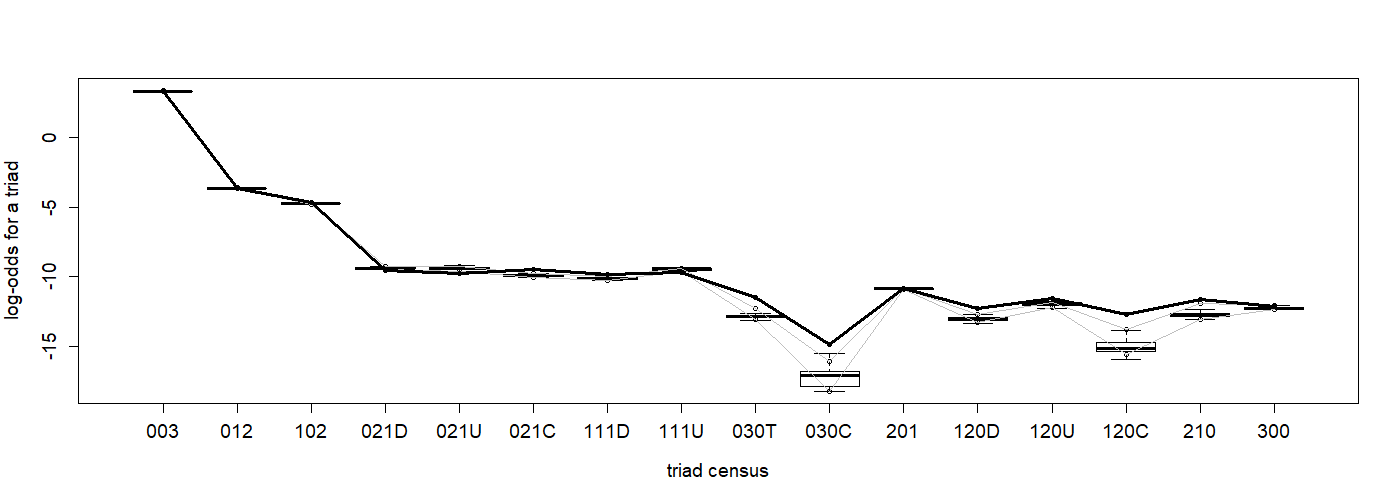 | |

Table S26. Correlation matrix and VIFs for the Time 1 cyber aggression network from the Long Island Study

|  | **Correlations** | | | |  | **VIFs** |
| --- | --- | --- | --- | --- | --- | --- |
|  | Edges | Mutual | GWI | GWO |  |  |
| Edges | 1.000 |  |  |  |  |  |
| Mutual | 0.638 | 1.000 |  |  |  | 1.013 |
| GWI | 0.959 | 0.599 | 1.000 |  |  | 1.006 |
| GWO | 0.984 | 0.613 | 0.946 | 1.000 |  | 1.012 |

Table S27. Goodness of fit summary for the Time 1 cyber aggression network from the Long Island Study

| 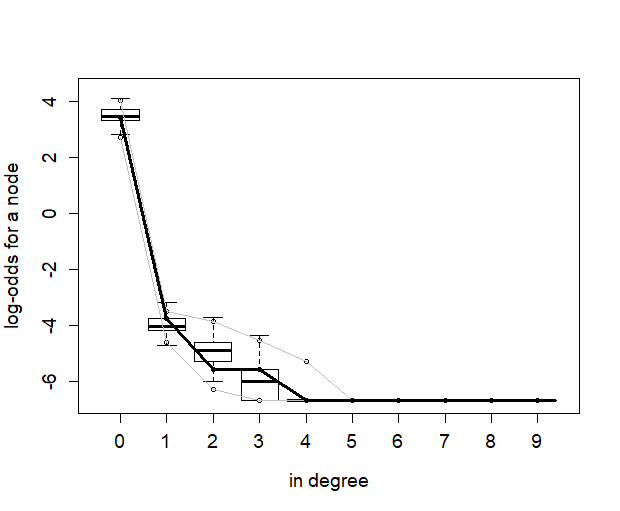 | 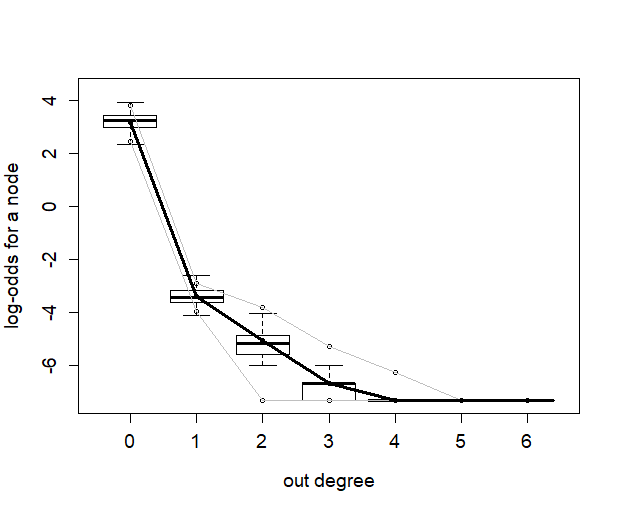 |
| --- | --- |
| 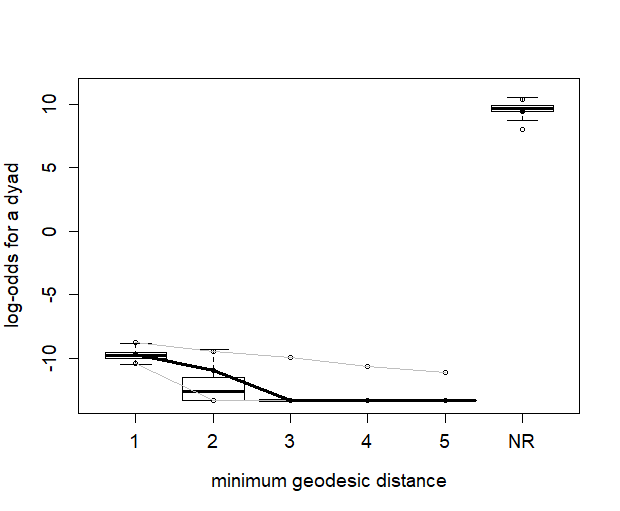 | 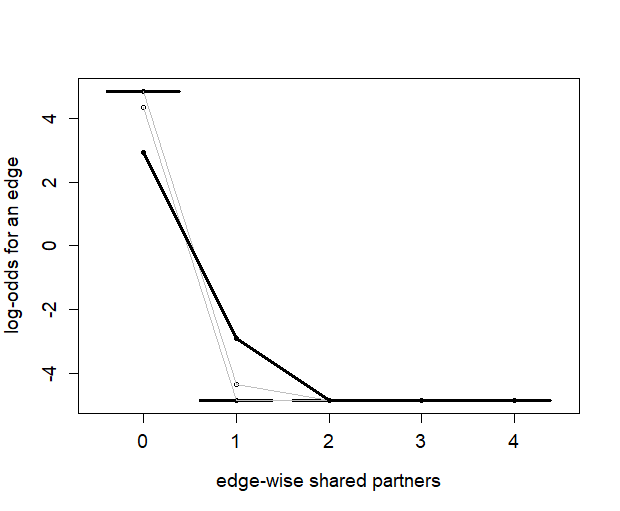 |
| 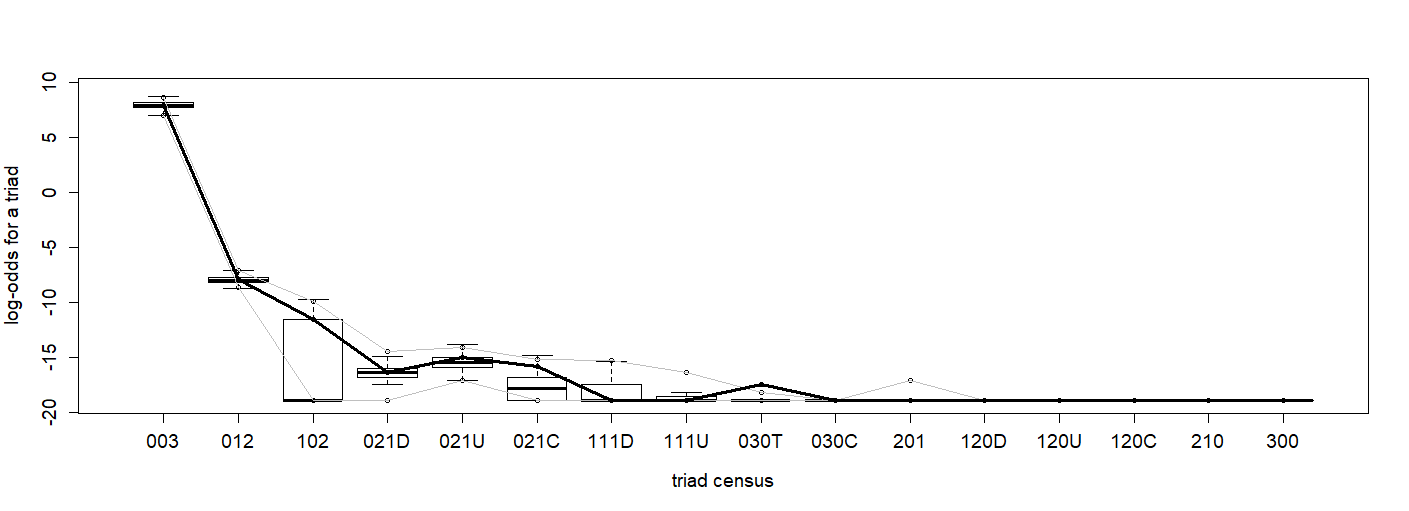 | |

Table S28. Correlation matrix and VIFs for the Time 1 Bitcoin OTC trust network

|  | **Correlations** | | | | |  | **VIFs** |
| --- | --- | --- | --- | --- | --- | --- | --- |
|  | Edges | Mutual | GWESP | GWI | GWO |  |  |
| Edges | 1.000 |  |  |  |  |  |  |
| Mutual | 0.954 | 1.000 |  |  |  |  | 1.767 |
| GWESP | 0.932 | 0.952 | 1.000 |  |  |  | 1.726 |
| GWI | 0.477 | 0.321 | 0.306 | 1.000 |  |  | 1.407 |
| GWO | 0.738 | 0.695 | 0.620 | 0.407 | 1.000 |  | 1.106 |

Table S29. Goodness of fit summary for the Time 1 Bitcoin OTC trust network

| 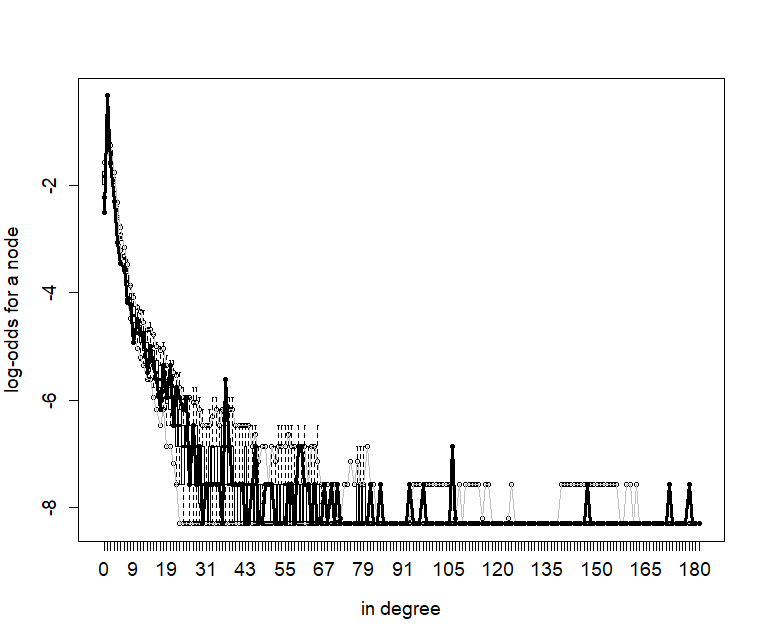 | 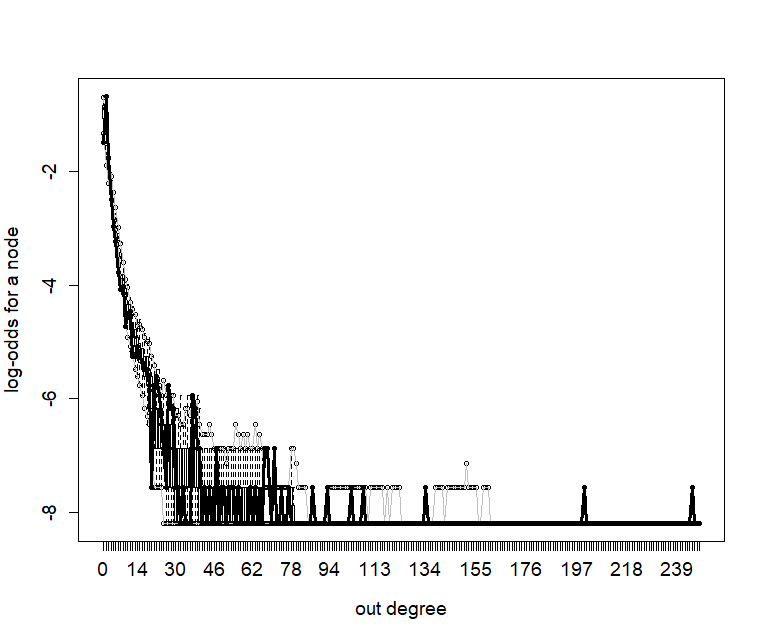 |
| --- | --- |
| 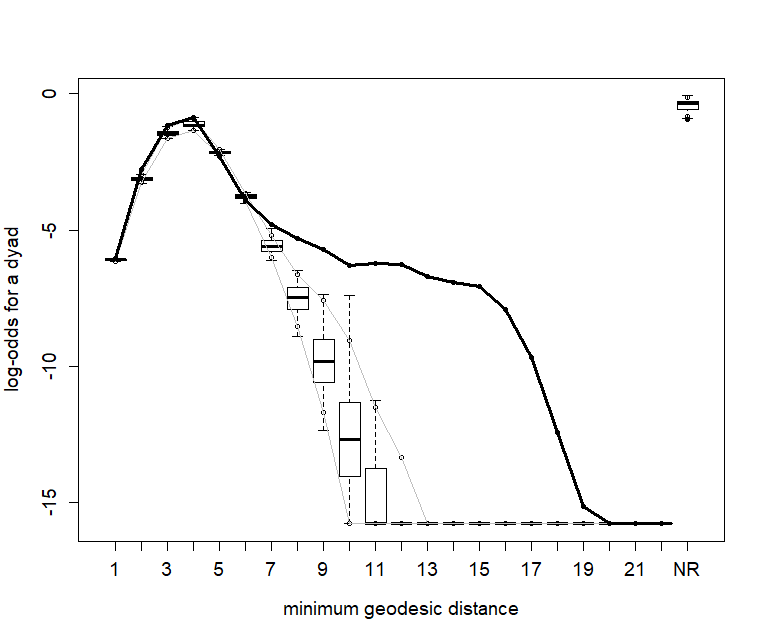 | 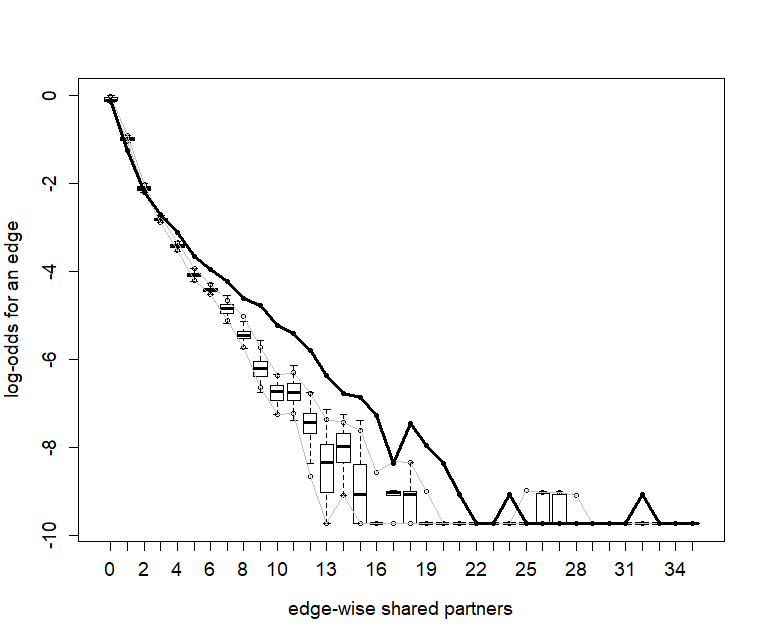 |
| 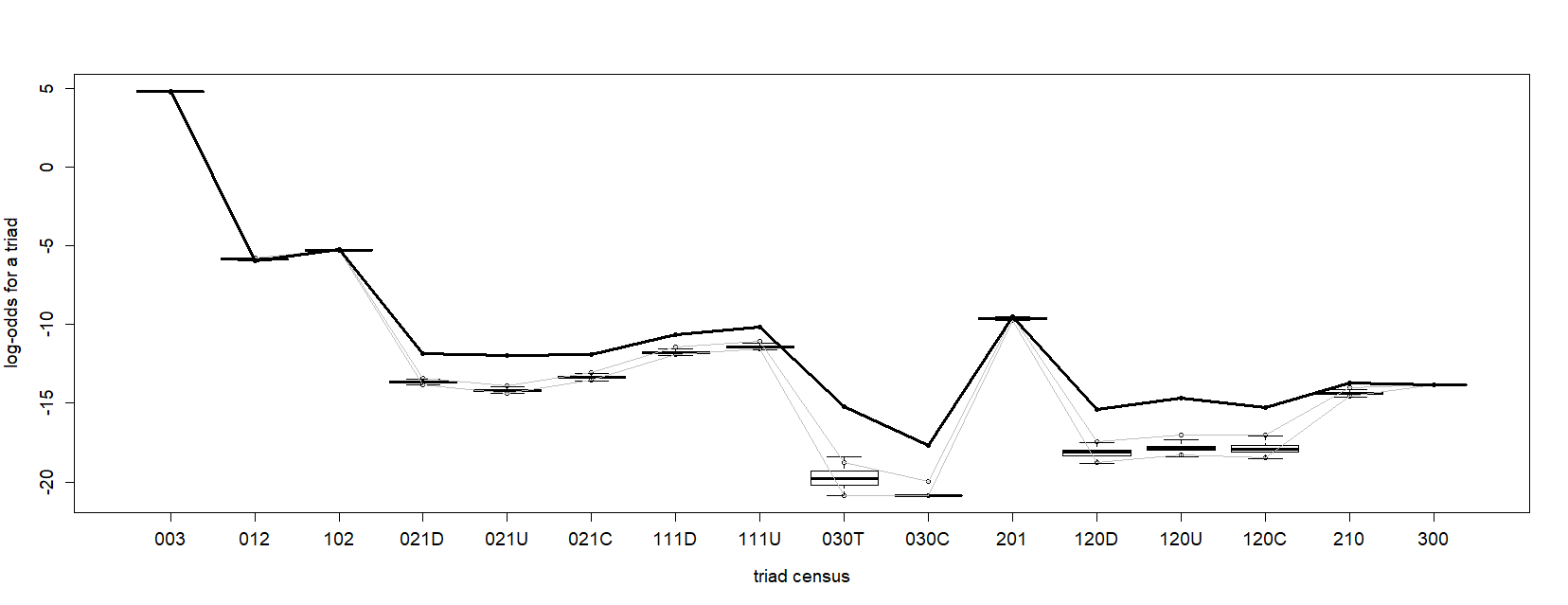 | |

Table S30. Correlation matrix and VIFs for the Time 1 Bitcoin OTC distrust network

|  | **Correlations** | | | |  | **VIFs** |
| --- | --- | --- | --- | --- | --- | --- |
|  | Edges | Mutual | GWESP | GWI |  |  |
| Edges | 1.000 |  |  |  |  |  |
| Mutual | 0.703 | 1.000 |  |  |  | 1.035 |
| GWESP | 0.020 | -0.054 | 1.000 |  |  | 1.011 |
| GWI | 0.918 | 0.691 | -0.004 | 1.000 |  | 1.028 |

Table S31. Goodness of fit summary for the Time 1 Bitcoin OTC distrust network

| 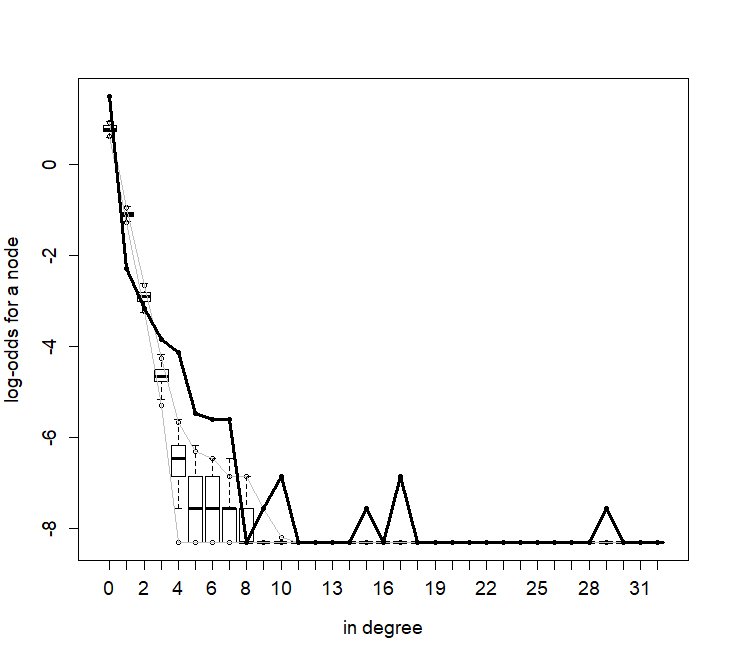 | 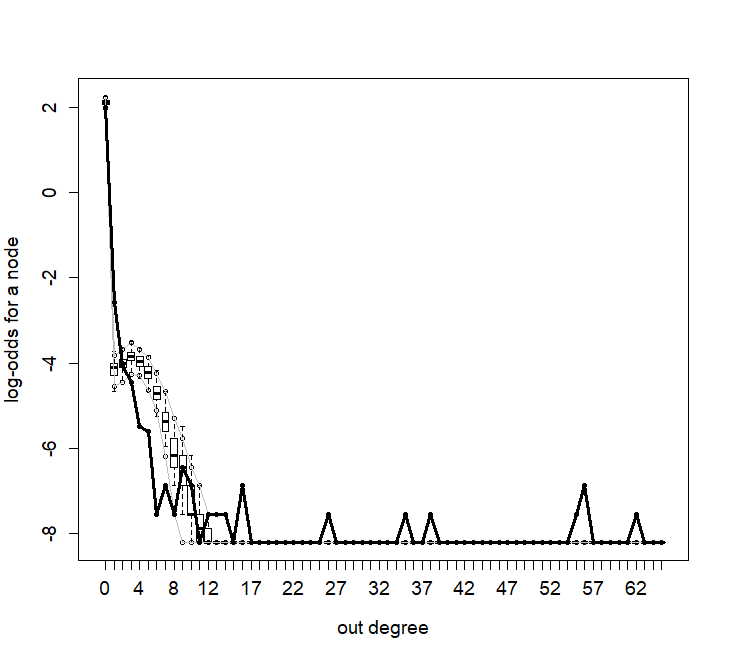 |
| --- | --- |
| 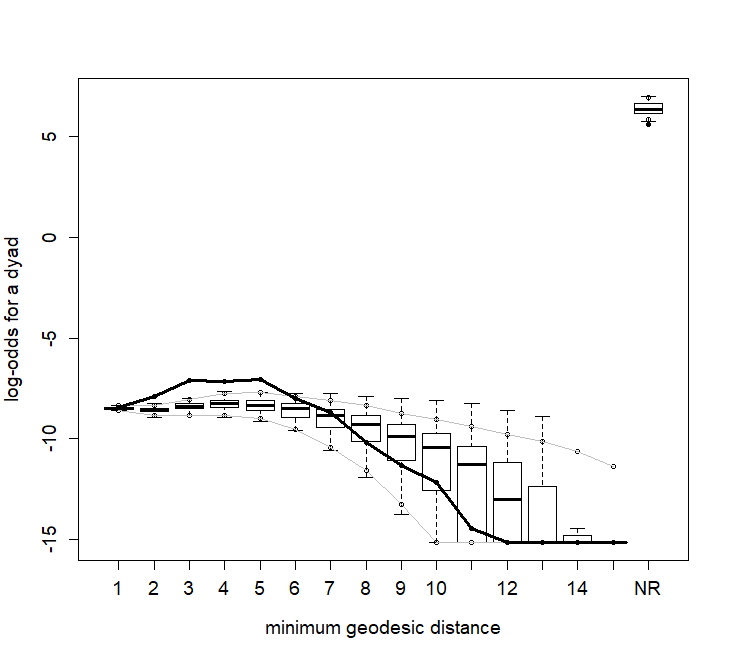 | 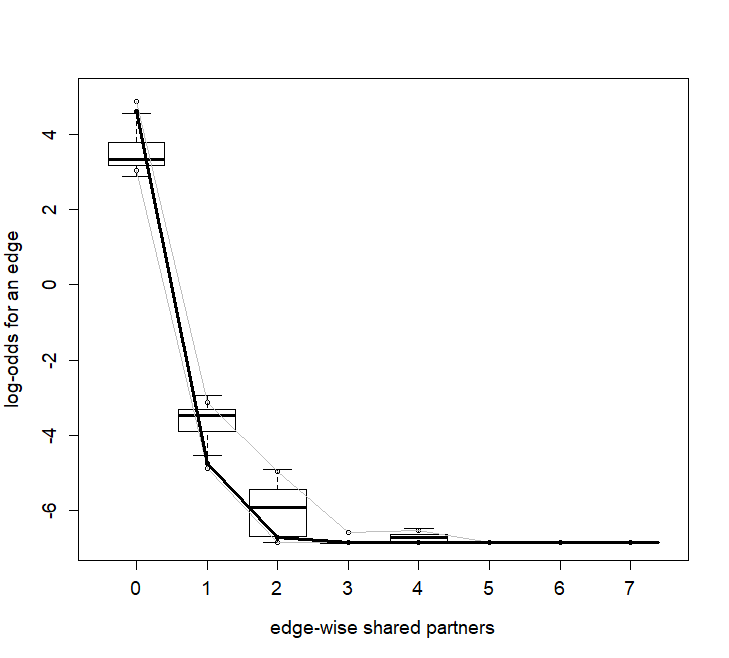 |
| 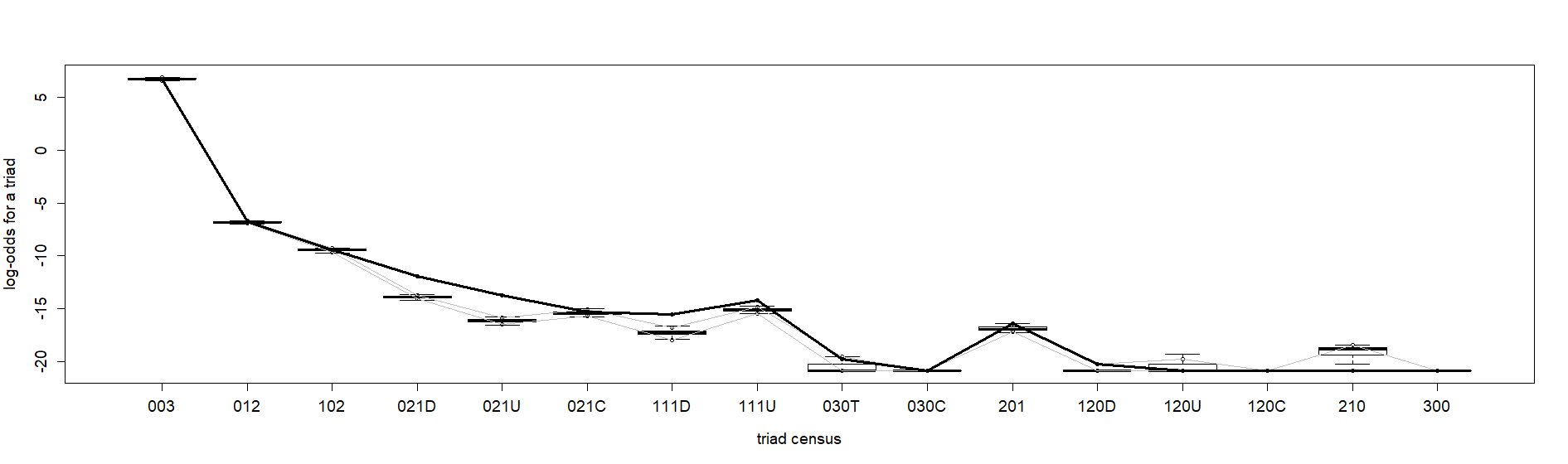 | |
